# IDLV-HIV-1 Env vaccination in non-human primates induces affinity maturation of antigen-specific memory B cells

**DOI:** 10.1038/s42003-018-0131-6

**Published:** 2018-09-05

**Authors:** Maria Blasi, Donatella Negri, Celia LaBranche, S. Munir Alam, Erich J. Baker, Elizabeth C. Brunner, Morgan A. Gladden, Zuleika Michelini, Nathan A. Vandergrift, Kevin J. Wiehe, Robert Parks, Xiaoying Shen, Mattia Bonsignori, Georgia D. Tomaras, Guido Ferrari, David C. Montefiori, Sampa Santra, Barton F. Haynes, Michael A. Moody, Andrea Cara, Mary E. Klotman

**Affiliations:** 1 Department of Medicine, Duke University Medical Center, Durham, 27710 NC USA; 2 Duke Human Vaccine Institute, Duke University Medical Center, Durham, 27710 NC USA; 3 Istituto Superiore di Sanità, Rome, 00161 Italy; 4 Department of Surgery, Duke University Medical Center, Durham, 27710 NC USA; 5 Department of Pathology, Duke University Medical Center, Durham, 27710 NC USA; 6 Beth Israel Deaconess Medical Center, Boston, 02215 MA USA; 7 Department of Pediatrics, Duke University Medical Center, Durham, 27710 NC USA

## Abstract

HIV continues to be a major global health issue. In spite of successful prevention interventions and treatment methods, the development of an HIV vaccine remains a major priority for the field and would be the optimal strategy to prevent new infections. We showed previously that a single immunization with a SIV-based integrase-defective lentiviral vector (IDLV) expressing the 1086.C HIV-1-envelope induced durable, high-magnitude immune responses in non-human primates (NHPs). In this study, we have further characterized the humoral responses by assessing antibody affinity maturation and antigen-specific memory B-cell persistence in two vaccinated macaques. These animals were also boosted with IDLV expressing the heterologous 1176.C HIV-1-Env to determine if neutralization breadth could be increased, followed by evaluation of the injection sites to assess IDLV persistence. IDLV-Env immunization was associated with persistence of the vector DNA for up to 6 months post immunization and affinity maturation of antigen-specific memory B cells.

## Abstract (web summary)

Maria Blasi et al. report the anti-HIV-1 humoral response elicited in rhesus macaques following vaccination with an SIV-based integrase-defective lentiviral vector (IDLV). They find that a single IDLV-Env immunization induces continuous antibody avidity maturation and boosting with a heterologous HIV-1 Env results in lower peak antibody titers than autologous boost.

## Introduction

The HIV-1 epidemic accounts for approximately 1.8 million new infections every year, and a growing number of recombinant vectors and DNA-based immunization strategies are actively being pursued as HIV-1 candidate vaccine platforms. However, some of these vaccine platforms are poorly immunogenic when administered alone^1^, recall pre-existing anti-vector immunity that can limit efficacy^2^, and to date have elicited short-lived immune responses^3^. Integrase-defective lentiviral vectors (IDLVs) are an alternative platform for vaccine development that can efficiently transduce both dividing and non-dividing cells and stimulate potent and durable antigen-specific immune responses^4–8^. Because of their combined immunogenicity and safety features, IDLVs are currently in development as vaccine platforms for anti-cancer therapy. Preliminary results from a human vaccine trial for solid cancers demonstrated safety and immunogenicity with early evidence of anti-tumor activity^9,10^. Another interesting feature that makes IDLV an attractive vaccine platform is the possibility of using a vesicular stomatitis virus G protein (VSV-G) serotype exchange strategy to reduce anti-vector immunity across multiple immunizations^11^.

We have recently shown in non-human primates (NHPs) that a single immunization with IDLV induced functional and durable (up to 1 year) antigen-specific immune responses that were strongly boosted by a second dose of the same vector^5^. In the present study we have assessed the effect of a single IDLV containing a heterologous envelope (Env) as a boosting injection in the same cohort of vaccinated NHPs and have analyzed both antibody affinity maturation and antigen-specific memory B-cell persistence. To determine whether the prolonged immune responses induced by IDLV correlated with the persistence of the vector in the muscle of the vaccinated animals, we biopsied the injection site and evaluated the presence of vector DNA and RNA by PCR. We found that IDLV immunization induced continued antibody affinity maturation 3 months post prime, with additional affinity maturation after the second IDLV immunization. HIV-1 1086.C gp140 Env-specific memory B cells persisted in the circulation for up to 8 months post prime, and vector DNA was still present in the muscle 6 months after the final IDLV-Env boost. Our results support the further development of IDLV-Env-based vaccination strategies for the elicitation of durable immune responses against HIV-1.

## Results

### Durable Env-specific Ab responses post IDLV-Env immunization

**Fig. 1:**
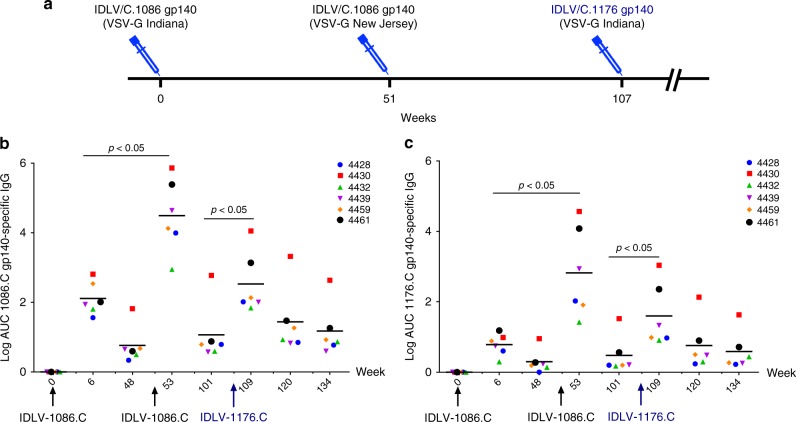
NHP immunization schedule and binding antibody responses. **a** Six Indian rhesus macaques were primed intramuscularly with 3 × 10^8^ transducing units (TUs) of IDLV-1086.C and boosted twice, at 1-year intervals, first with the same vector IDLV-1086.C and then with IDLV expressing a different envelope 1176.C in an attempt to garner neutralization breadth. Anti-vector immunity in the repeated IDLV immunizations was minimized using two different VSV serotypes for the first and second immunizations. **b** Anti C.1086 gp140 and **c** anti 1176.C gp140 protein binding Abs induced by IDLV-Env immunization. The magnitude and durability of anti-Env IgG were measured in 6 animals in plasma. No binding response was detected in plasma samples collected before immunization (week 0). Lines indicate mean values at each time point. Each sample was analyzed in duplicate and the data shown are representative of at least three experiments

Six Indian rhesus macaques were immunized intramuscularly with IDLV expressing the 1086.C (weeks 0 and 51) and the 1176.C envelopes (week 107) as shown in Fig. 1a. Plasma antibodies (Abs) specific for 1086.C-Env or 1176.C-Env were assessed at 2 weeks post immunization and then monthly thereafter. The data in Fig. 1b, c were assessed in a single assay to reduce the contribution of inter-assay variability. We included previously tested time points for comparison^5^. As previously shown^5^, all NHPs developed high titers of 1086.C gp140 Env-specific Abs at 6 weeks post prime (Fig. 1b) that were strongly boosted by the week 51 immunization. IDLV-1176.C immunization at week 107 resulted in an increase in Ab titers compared to week 101 (1 year post second immunization) (*p* = 0.03125 by Wilcoxon signed-rank test), but Ab titers were lower compared to the peak at week 53. Humoral responses waned over time, but were still detectable 1 year after the first and second immunizations and at least to 27 weeks following the third immunization. We performed enzyme-linked immunosorbent assays (ELISAs) with the 1176.C gp140 protein to determine whether Abs induced by IDLV-Env immunization could bind both 1176.C and 1086.C envelopes. As shown in Fig. 1c, binding responses against the 1176.C gp140 were lower than against the 1086.C gp140, suggesting that some of the Abs induced by IDLV-1086.C were directed towards epitopes not present on the 1176.C gp140. However, similar to the binding Ab responses to 1086.C, Abs able to bind 1176.C gp140 were boosted by the second IDLV-1086.C immunization and more weakly by the IDLV-1176.C immunization. Interestingly, IDLV-1176.C immunization resulted in higher titers of 1086.C-reactive Abs than 1176.C-reactive Abs, suggesting a preferential engagement of cross-reactive clones that were primed by IDLV-1086.C.

**Fig. 2:**
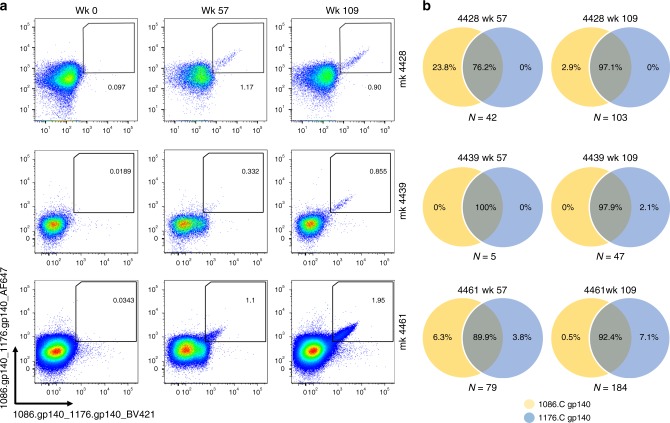
Cross-reactivity of memory B cell-derived antibodies from longitudinal samples against 1086.C and 1176.C gp140 Envs. **a** Flow plots of 1086.C and 1176.C gp140 staining of Env-reactive memory B cells in the indicated animals at 0, 57, and 109 weeks post immunization. Memory B cells which bound both BV421 (*x*-axis) and AF647 (*y-*axis) labeled Envs were defined as double positive (DP) Env-reactive memory B cells. Sorting of DP Env-reactive B cells was performed to ensure sort accuracy. No pre-existing cross-reacting memory B cells were present before vaccination (week 0). **b** Env reactivity of each individual sorted memory B cell was confirmed in ELISA after in vitro expansion and differentiation into antibody-secreting cells as described in the Methods. The Venn diagrams show the percentage of Env-specific supernatants from Ig-secreting cultured memory B cells that bound to 1086.C gp140 (yellow) and/or 1176.C gp140 (blue) Env. The number of Env-specific memory B-cell supernatants screened for each animal at each time point is indicated below each diagram

To assess the cross-reactivity of individual Abs from the memory B-cell compartment, we sorted 1086.C- and 1176.C-reactive memory B cells from peripheral blood taken at weeks 57 and 109 post immunization from 3 out of 6 animals (Fig. 2a) and cultured them at limiting dilution (1 cell per well) for 2 weeks to induce their differentiation into antibody-secreting cells^12^. After in vitro stimulation, each culture supernatant was individually tested for binding to 1086.C and 1176 gp140 Envs in ELISA. To rule out pre-existing immunity against the two antigens, we also assessed the presence of antigen-specific memory B cells on peripheral blood mononuclear cells (PBMCs) collected before immunization (week 0) (Fig. 2a). The frequency of 1086.C and/or 1176.C Env gp140-specific memory B cells over the total pool of circulating memory B cells ranged from 0.3 to 1.2% at week 57 and from 0.9% to 2% at week 109 (Fig. 2a). The majority of supernatants from Env-specific memory B cells collected at week 57 (6 weeks post IDLV-1086.C immunization) produced IgG antibodies that reacted with both 1086.C and 1176.C gp140 Envs (range: 76.2–100%), as predicted by the Ab serum data (Fig. 2b). Three culture supernatants from NHP 4461 bound only to 1176.C gp140 Env, suggesting that these Abs have better affinity for the heterologous Env. Following the IDLV-1176.C immunization (week 109), >90% of the isolated memory B cells bound to both 1086.C and 1176.C gp140 Envs (range: 92.4–97.9%) (Fig. 2b); the frequency of memory B cells that reacted with only 1086.C Env decreased 10-fold from week 57 (from 23.8% to 2.9% in NHP 4428 and from 6.3 to 0.5% in NHP 4461), whereas only a small fraction of memory B cells bound only to 1176.C gp140 Env (range: 0–7.1%) (Fig. 2b). These data demonstrate that IDLV-1086.C induced memory B cells that mostly cross-reacted with 1176.C gp140 Env, and the IDLV-1176.C boost did not establish a large pool of memory B cells that bound only to 1176.C Env.

### Detection of anti-VSV-G neutralizing Abs in serum

To reduce anti-vector immunity from repeated IDLV injections, IDLV particles were pseudotyped with VSV glycoproteins from the Indiana serotype (first and third injections) and the New Jersey serotype (second injection). It has been previously shown that despite inducing autologous neutralizing antibodies (nAbs), these envelopes did not induce cross-neutralizing antibodies to the other serotype^11^. To evaluate VSV-G serum nAbs induced after each of the three IDLV immunizations, we performed a neutralization assay using green fluorescent protein (GFP)-expressing IDLV pseudotyped with the two different VSV-G serotypes on serum samples from all six animals. Vaccinated NHPs developed nAbs against the Indiana-G serotype 2 weeks post prime that rapidly declined to baseline levels between weeks 6 and 14. The New Jersey-G serotype induced much lower nAbs against itself and no cross-neutralization of the Indiana serotype (Table 1) was detected. As expected, we detected a boost in nAbs against the Indiana-G serotype following the third immunization. These boosted nAbs waned more slowly when compared to the first injection but cross-neutralization of the New Jersey-G serotype was not detected. Serum from animal 4430 was tested against an additional VSV-G serotype not used in this study, Cocal-G, and we found low levels of cross-neutralization 2 weeks after each immunization. Our findings indicate that there is little cross-neutralization between these three VSV-G serotypes and the induction of low levels on nAbs against the New Jersey serotype suggests that, in the context of prolonged intervals between IDLV immunizations, this same VSV-G serotype can be used to deliver the target immunogen without a loss of vaccine potency.

**Table 1:** Anti-VSV-G nAb titers following each IDLV immunization

Animal ID	4428		4430			4432		4439		4459		4461	
Week	Indiana	New Jersey	Indiana	New Jersey	Cocal	Indiana	New Jersey	Indiana	New Jersey	Indiana	New Jersey	Indiana	New Jersey
0	0	0	0	0	0	0	0	0	0	0	0	0	0
**2**	**>1000** **<** **2000**	**0**	**>1000** **<** **2000**	**0**	**<500**	**<500**	**0**	**>1000** **<** **2000**	**0**	**>2000**	**0**	**>2000**	**0**
6	<500	0	<500	0		<500	0	<500	0	2000	0	>1000 < 2000	0
14	<500	0	<500	0		0	0	<500	0	>500 < 1000	0	>500 < 1000	0
**53**	**<500**	**<500**	**<500**	**<500**	**<500**	**0**	**0**	**<500**	**0**	**<500**	**<500**	**>1000** **<** **2000**	**<500**
57	0	0	0	0		0	0	<500	0	N/A	N/A	>500 < 1000	N/A
64	0	0	0	0		0	0	<500	0	<500	0	<500	0
**109**	**>1000** **<** **2000**	**0**	**>1000** **<** **2000**	**0**	**<500**	**>500** **<** **1000**	**0**	**>2000**	**0**	**>2000**	**0**	**>2000**	**0**
113	>500 < 1000	0	>500 < 1000	0		500	0	>1000 < 2000	0	>1000 < 2000	0	>2000	0
120	>500 < 1000	0	>500 < 1000	0		>500	0	>500 < 1000	0	>1000 < 2000	0	>1000 < 2000	0
124	0	N/A	>500 < 1000	N/A		>500	0	500	0	>1000 < 2000	0	>1000 < 2000	0
128	0	N/A	>500 < 1000	N/A		>500	0	<500	0	>1000 < 2000	0	>1000 < 2000	0

Anti-VSV-G nAb titers are expressed as the highest dilution of serum that results in 50% reduction in fluorescence when compared to vector treated with week 0 sera. The bold lines indicate the peak antibody responses post each immunization. 1:500 was the lowest dilution tested. Values of <500 indicate that a reduction in the percentage of GFP-positive cells was detected, but the reduction was lower than 50%. Values = 0 indicate that no reduction in GFP expression was observed at the indicated time points

### Antibody affinity maturation after a single IDLV injection

**Fig. 3:**
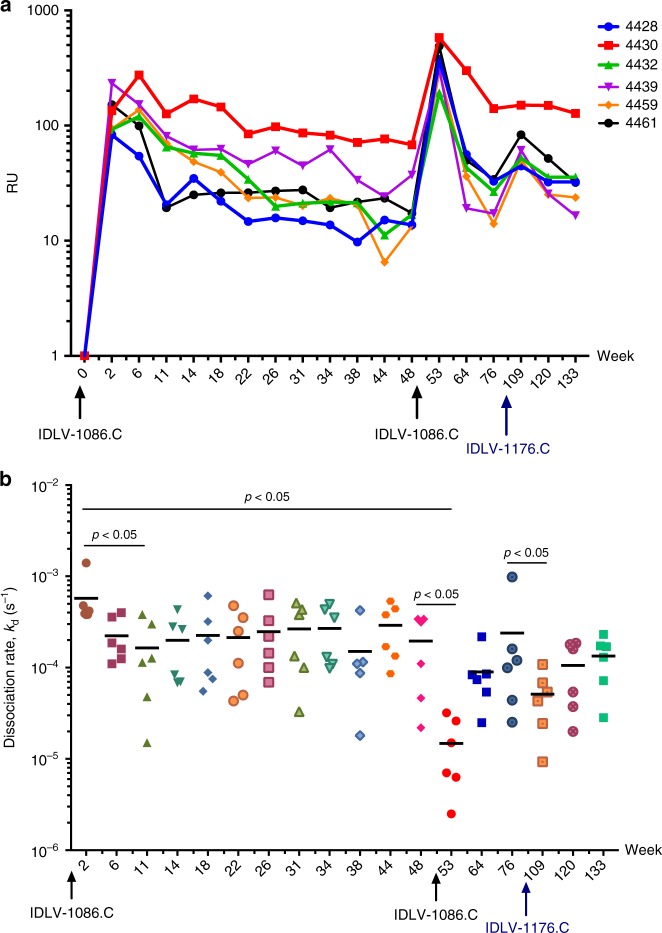
IDLV vaccination induces antibody avidity maturation over time. Purified IgG was isolated from serum and avidity for 1086.C gp120 was tested in SPR. **a** Binding in response units (RUs) to 1086.C Env gp120 protein over time. **b** Antibody dissociation rate (*k*_d_ in s−1) over time. Data shown are average of two experiments. Lines indicate mean values at each time point

To assess whether a single IDLV immunization induced antibody affinity maturation, we purified IgG from serum and measured Ab binding responses and avidity to 1086.C gp120 via surface plasma resonance (SPR). Binding to 1086.C Env gp120 was detected at week 2 after the prime, and while responses declined over time in the majority of animals, all IgG samples retained binding up to week 48 after the first immunization (Fig. 3a). From week 2 to week 11, the dissociation rate (*k*_d_) gradually decreased over time, indicating more avid Ab binding to 1086.C gp120 (*p* = 0.03125 by Wilcoxon signed-rank test). The median *k*_d_ plateaued and was sustained through week 48. Boosting with IDLV-1086.C resulted in both higher binding responses (Fig. 3a) and a dramatic reduction in *k*_d_ compared to that measured at 2 and 48 weeks post prime (*p* = 0.03125 by Wilcoxon signed-rank test) (Fig. 3b). This effect was transient and the *k*_d_ of serum 1086.C gp120-specific IgG returned to pre-boost levels after 6 months. These data suggest that an increase in avidity for gp120 continued for at least 3 months post prime, and could be further stimulated by a subsequent boost, although this effect was transient. The third immunization with IDLV-1176 boosted both gp120 binding and avidity (*k*_d_) compared to week 76 (pre-second boost), but neither response was as strong when compared to week 53 (post first boost).

### Epitope mapping

**Fig. 4:**
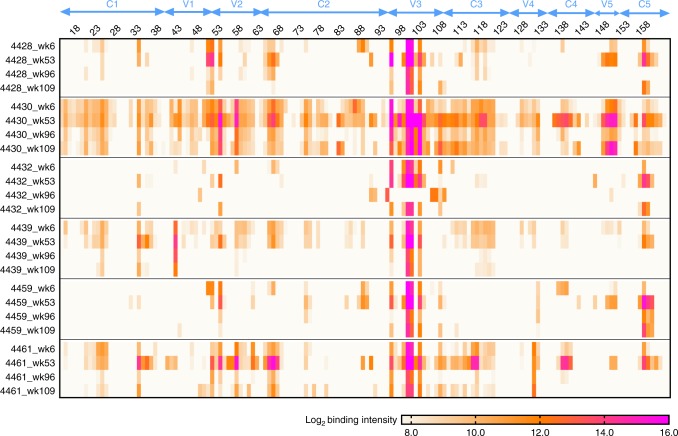
Linear epitope mapping. The heat map shows gp120 binding over time to multiple HIV-1 strains. Binding intensity is shown for each peptide, corrected with its own background value

To determine whether the IDLV-1176.C boost induced antibodies that recognized different linear epitopes than those induced by IDLV-1086.C, we performed a linear epitope mapping using peptide microarray covering a panel of Env sequences on serum samples from weeks 6, 53 (2 weeks post DLV-1086.C boost), 96 (45 weeks post IDLV-1086.C boost), and 109 (2 weeks post IDLV-1176.C boost) post prime. We have previously shown that IDLV-1086.C immunization induced a dominant and sustained cross-clade gp120-directed binding Ab response to the Env third variable loop (V3)^5^. In this study, we detected binding to a V2 hotspot (peptides 50–55 in Fig. 4)^13^ in all six NHPs, and 3/6 NHPs (4430, 4439, and 4461) bound to peptides at the V2.α4β7 site (peptides 56–58 in Fig. 4); these responses were highest at week 53 post prime (2 weeks post IDLV-1086.C boost). Immunization with IDLV-1176.C did not boost linear epitope binding responses and did not induce new linear specificities relative to the earlier responses to IDLV-1086.C (Fig. 4).

### Env-specific memory B-cell durability in vaccinated animals

**Fig. 5:**
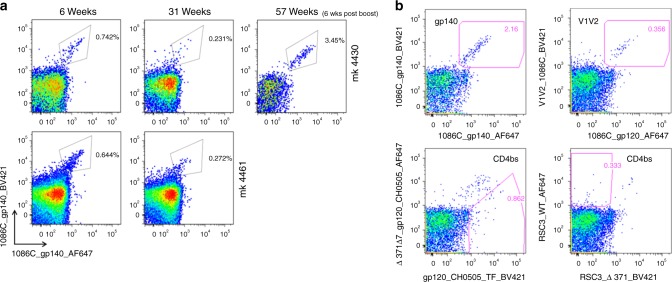
Persistence of antigen-specific circulating memory B cells following IDLV vaccination. **a** To assess the persistence of the 1086.C-specific circulating memory B cell pool, longitudinal flow cytometry analysis was performed on PBMCs from two vaccinated animals. **b** Flow plots of gp140 and gp120 (1086, CH505 or RSC3) staining of Env-reactive memory B cells in animal 4430 at 57 weeks post immunization. Memory B cells which bound both BV421 (*x*-axis) and AF647 (*y*-axis) labeled Envs were defined as double positive (DP) Env-reactive memory B cells. Sorting of DP Env-reactive B cells was performed to ensure sort accuracy. Memory B cells which bound to the wild-type form of gp120 but not to the d371 mutated Env were defined as differential binders (putative CD4bs Abs)

To assess whether a single IDLV immunization resulted in persistence of antigen-specific memory B cells in blood, we performed flow cytometry analysis on longitudinal PBMC samples from two vaccinated animals. Interestingly, a single IDLV injection induced 1086.C-specific memory B cells that circulated 31 weeks post prime and expanded after the second immunization 51 weeks post prime (Fig. 5a). Thus, an IDLV-based HIV-1 immunogen showed extended durability of the vaccine-induced memory B-cell pool.

### Genetic characteristics of Env-specific monoclonal Abs

To characterize vaccine-elicited Abs, we selected NHP 4430 for further analysis because of high antigen-specific IgG (Fig. 3a). We performed single-cell PCR on HIV-1-specific memory B cells sorted from peripheral blood taken at week 57. Our sort strategy was designed to isolate 1086.C gp140, 1086.C gp120 V1/V2, and CD4 binding site Abs (defined by binding to the wild-type CH505 gp120 but not to a version with a deletion at amino acid 371 that disrupts the CD4 biding site^14^) as shown in Fig. 5b. We isolated 80 monoclonal antibodies (mAbs) from NHP 4430 and confirmed HIV-1 Env reactivity by ELISA using clade B, C, and AE gp120 or gp140 proteins (Supplementary Data 1). We isolated 20 additional mAbs for which we could not measure Env reactivity because of their low level of expression in small-scale transfection (IgG concentration < 0.001 μg mL^−1^), and 6 mAbs that did not confirm envelope reactivity in ELISA. These 26 mAbs were excluded from further analysis. Sorting of double positive 1086.C gp140-specific memory B cells yielded 33 mAbs of which 85% bound to gp120 and 15% bound to gp41. These data suggest that the expression of the gp140 HIV-1 Env by IDLV did not induce a dominant gp41 antibody response as was sometimes observed in both human and NHP studies of Ad5 vector-based vaccine regimens^15,16^. Isolated gp120-reactive mAbs included V3, V2, and CD4bs-directed specificities (Supplementary Data 1). Immunogenetic analysis of all paired heavy and light chains was conducted using Cloanalyst to determine V_H_ and V_L_ gene usage, complementarity-determining region 3 (CDR3) length, and mutation frequency^17^. Of the 80 mAbs, 46 (57%) utilized gene segments from the Rhesus IGHV4 family. Interestingly, we identified seven B-cell clonal lineages, each comprised of two or three mAbs: five lineages were V2 specific, one lineage was CD4bs specific, and one lineage was V3 specific (Supplementary Table 1). The median heavy chain CDR3 (HCDR3) length was 16 amino acids and the mean V_H_ mutation frequency was 8%. Fourteen mAbs representative of the diverse specificities (Supplementary data 1) were selected for large-scale production to evaluate their ability to mediate neutralization and antibody-dependent cell-mediated cytotoxicity (ADCC). We measured neutralization against five clade C viruses (Table 2) and ADCC activity against two clade C viruses (MW965.25 and 1086.C). Six mAbs (42.9%) neutralized tier 1 virus MW965.26 with half-maximal inhibitory concentration (IC_50_) ranging from <0.02 to 26.6 µg mL^−1^ (Table 2). Mab 911901 also neutralized tier 2 heterologous HIV strain 25710-2.43 (IC_50_: 13.5 µg mL^−1^). These data demonstrate that the vaccine induced memory B cells producing antibodies that neutralize tier 1 strains. None of the tested mAbs mediated ADCC against target cells infected with the autologous 1086.C virus or the MW965.25 virus.

**Table 2:** mAb neutralization assay against the indicated viruses

		Neutralization panel				
Ab ID	Binding specificity	SVA-MLV (Neg. ctrl)	MW965.26 (tier 1)	Ce1086_B2 (tier 2)	Ce1176_A3 (tier 2)	25710-2.43 (tier 2)
910893	V1V2	>50	>50	>50	>50	>50
910894	CD4bs	>50	>50	>50	>50	>50
910895	V1V2	>50	>50	>50	>50	>50
910900	V1V2	>50	>50	>50	>50	>50
911883	V1V2	>50	>50	>50	>50	>50
911884	V1V2	>50	>50	>50	>50	>50
911890	gp120	>50	**0.05**	>50	>50	>50
911892	V3	>50	**<0.02**	>50	>50	>50
911897	CD4bs	>50	**0.89**	>50	>50	>50
911899	CD4bs	>50	>50	>50	>50	>50
911900	gp120	>18.5	**0.57**	>18.5	>18.5	>18.5
911901	V3	>50	**<0.02**	>50	>50	**13.53**
911904	CD4bs	>50	**26.59**	>50	>50	>50
911905	V1V2	>50	>50	>50	>50	>50

Values are the antibody concentration in µg mL^−1^ at which relative luminescence units (RLUs) were reduced 50% compared to virus control wells (no test sample) after subtraction of background RLUs in cell control wells. A response was considered positive if the IC_50_ was 3 times greater than the signal against the MLV-pseudotyped negative control virus. The bold values indicate a positive response. The binding specificity on the HIV-1 envelope is shown for each mAb

### Lack of neutralization breadth increase post IDLV-1176 boost

We previously showed that prime and boost with IDLV-1086.C gp140 alone did not result in neutralization breadth^5^, and thus we performed a heterologous boost with IDLV expressing the 1176.C gp140 Env^18^ in an attempt to broaden the response. The 1176.C gp140 was selected because it possesses neutralization epitopes not present on the 1086.C envelope^19^, including epitopes targeted by many known broadly neutralizing antibodies (bnAbs) such as glycans at position N160^20^ and N332^21^. At 2 weeks after the IDLV-1176 injection, we detected an increase in the neutralizing antibody titers against the tier 1 virus MW965.26 (Supplementary Table 2). However, no tier 2 HIV-1 neutralization was observed.

### Persistence of IDLV DNA and transgene expression over time

**Fig. 6:**
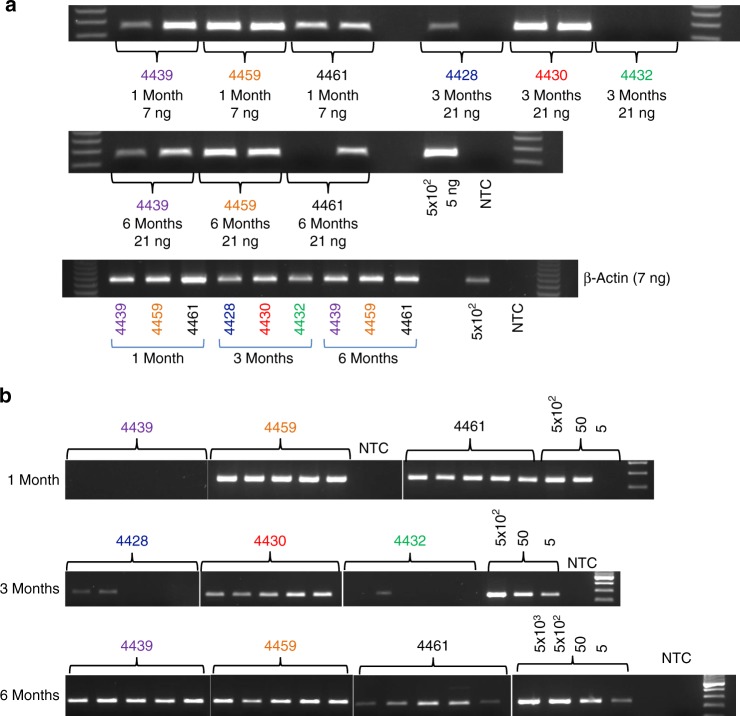
IDLV DNA persists at the site of injection at 6 months post immunization. **a** Total-IDLV DNA PCR and **β**-actin PCR were performed using the indicated DNA amounts extracted from the muscle biopsies. Genomic DNA (5 ng) extracted from the positive control (CMMT-LV-Neo) corresponding to 5 × 10^2^ cells was also amplified. **b** Five PCR replicates were performed for each sample to increase the probability of detecting IDLV DNA in all the animals

To assess whether the durability of the immune response to IDLV correlated with vector persistence and prolonged transgene expression, we collected muscle biopsies after the last IDLV-1176.C immunization. At 1 month before IDLV-1176.C injection, we tattooed injection site targets on the thighs of the NHPs bilaterally. The tattoo allowed us to trace more precisely the injection sites and excise small tissue biopsies at different time points post injection (Table 3). At 2 weeks and 1, 3, and 6 months after injection, injection site muscle biopsies were assayed for retro-transcribed vector DNA by PCR. IDLV DNA was detected in all animals at every assayed time point post immunization (Fig. 6a, b). We also evaluated transgene expression by reverse transcription polymerase chain reaction (RT-PCR) but were not able to detect vector RNA in any of the muscle biopsies tested. This negative result may be due to degradation of RNA during handling or due to low RNA yields as assessed by β-actin PCR (Supplementary Figure 1). To further assess this question, we performed intramuscular immunizations of four mice with GFP-expressing IDLV, followed by extraction of RNA from whole thigh muscles 3 months post injection from which we were able to amplify IDLV RNA (Supplementary Figure 2). In combination, our data indicate that IDLV DNA and RNA expression can persist in muscle tissue consistent with continuous antigen expression.

**Table 3:** Muscle biopsies schedule

	**Animal ID**	**Weeks post IDLV-1176 boost**	
Group A	4428	2	12
	4430	2	12
	4432	2	12
Group B	4439	4	24
	4459	4	24
	4461	4	24

Monkeys were tattooed before the third IDLV-1176 gp140 immunization in order to be able to find the injection sites and muscles biopsies were then performed at 2 weeks and at 1, 3, and 6 months post immunization on 3 monkeys per time point, sampling 1 thigh per monkey as indicated in the table

**Fig. 7:**
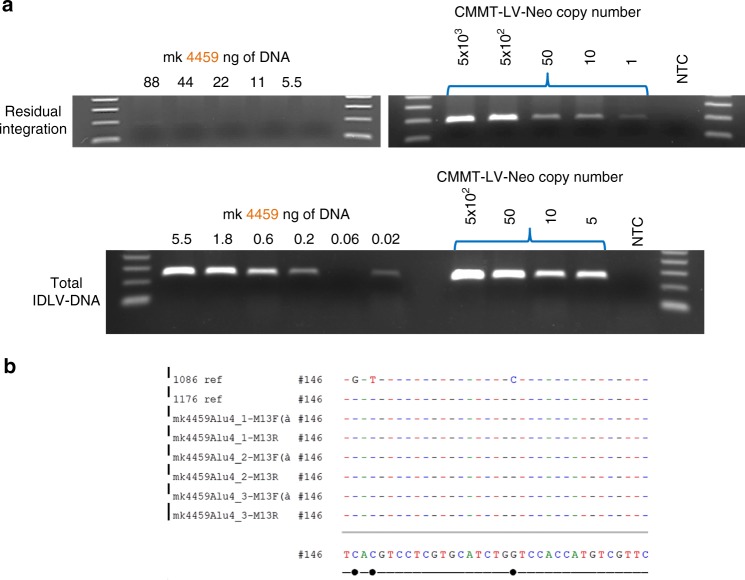
Detection of residual integration in NHP 4459 at 1 month post IDLV-1176 immunization. **a** Alu-PCR for vector integration and total-IDLV PCR were performed on serial dilutions of total DNA extracted from 1-month muscle biopsy of NHP 4459 (left) and genomic DNA extracted from CMMT-LV-Neo positive control (right). **b** Positive bands in Alu-PCR were extracted and cloned into pCR2.1 TOPO vector and subsequently sequenced using the M13F and M13R primers. The sequence alignment shows that the integrated sequence corresponds to IDLV-1176

While the frequency of IDLV integration is markedly reduced compared to integrase-competent lentiviral vectors, low levels of non-integrase-mediated integration have been observed^22–26^. To determine whether we could detect residual integration at the injection site, we performed an Alu-PCR using the primers and conditions in Supplementary Table 3. Residual integration was detected in only one out of six animals at 1 month post IDLV-1176.C immunization (Fig. 7), but integrated vector DNA could not be detected in the 6-month biopsy sample in the same animal (data not shown). To determine the rate of residual integration relative to the total amount of vector DNA present in the 1-month muscle biopsy from animal 4459, we performed Alu-PCR and total-IDLV PCR on serial dilutions of the sample. Vector DNA was still detectable in as little as 20 pg of total DNA, while residual integration was not detected in samples with less than 44 ng of total DNA (Fig. 7a). We thus calculated the rate of IDLV integration at about 1 integration event per >2000 copies of vector DNA. Our results are in line with previously reported data. To identify the integrated vector, we cloned and sequenced the amplified PCR product showing that the integrated DNA arose from IDLV-1176 (Fig. 7b).

## Discussion

Although several viral vectors are being explored in efforts to design a potential HIV-1 vaccine, adenovirus and poxvirus-based vectors have received the most attention^27^. Adenoviral vectors have been shown to be highly immunogenic and to induce robust cytotoxic T lymphocyte responses but their effectiveness has been limited by pre-existing host immunity^28^, and vaccine regimens based on Ad5 showed no protective efficacy in the STEP, Phambili, or HVTN 505 studies^29^. Conversely, a canarypox-based vector employed in the RV144 trial provided a moderate 31% protection^30^ but the humoral immune response waned rapidly after vaccination^31^. Thus, there is a need for vaccine regimens that provide more durable responses and that lack the problems of pre-existing vector immunity. Integrase-defective lentiviral vectors are a promising antigen delivery system given their ability to induce both robust and durable immune responses to engineered antigens in humans and animal models alike^5,6,10^.

In this study, we found that following a single immunization with IDLV expressing the C.1086 gp140 envelope, antigen-specific antibody responses underwent affinity maturation through 3 months post prime, and that the antibody avidity could be further enhanced by subsequent IDLV boosts, although this effect was transient. One hypothesis is that the second IDLV-1086.C immunization rapidly engaged the pool of memory B cells that was elicited by the first vaccination and promoted affinity maturation, which resulted in the dramatic change in *k*_d_ observed at week 53. Then, newly engaged naive B cells with suboptimal affinity emerged, which resulted in the increase in *k*_d_ observed at week 64. This hypothesis is supported by recent studies showing that B cells activated by complex antigens, like the HIV-1 envelope, establish germinal centers that are permissive for a diverse spectrum of affinity and clonality^32^. This permissive environment allows the expansion of lower affinity and/or polyreactive B cells that enter the memory B-cell pool and become potentially useful templates for later evolutionary refinement^33^.

Analysis of antigen-specific circulating memory B cells in two vaccinated animals demonstrated persistence to at least 8 months post prime, correlating with the durability of the antibody responses observed. Importantly, this IDLV regimen did not result in a gp41-dominant antibody response, a finding that contrasts with recent publications that showed a gp41-dominant response to immunogens containing gp41 in both humans and macaques due to cross-reactivity between gp41 and gut microbiota^15,16^. In contrast, both linear epitope mapping and single memory B-cell repertoire analysis on the IDLV-C.1086 gp140 vaccinated animals showed a dominant gp120 antibody response. This, together with the lack of measurable pre-existing immunity against the 1086.C and 1176.C gp140 Envs in pre-vaccination memory B cells, suggests that expression of the C.1086 antigen by IDLV did not boost pre-existing cross-reactive gp41-microbiota antibody responses. Serum mapping by linear epitope peptide array demonstrated a dominant V3-binding response but a larger number of mAbs isolated from antigen-specific memory B cells were specific for the V2 loop. While we cannot rule out bias induced by the sorting process, it is interesting to note that analysis of plasma from the RV144 HIV-1 vaccine efficacy trial showed a significant inverse correlate of risk associated with Ab reactivity with tags C.1086 V1V2^34^. Our recovery of V2-specific mAbs after immunization with IDLV expressing the C.1086 envelope suggests that this vaccine strategy might be able to improve on the observed 31% efficacy by enhancing anti-V2 antibody durability.

The induction of tier 2 neutralizing antibody responses following vaccination has proven challenging. We did not observe neutralization breadth after two immunizations with IDLV-1086, and thus we evaluated whether a third injection with IDLV expressing a heterologous envelope, 1176.C, could induce tier 2 neutralizing antibody responses. We did not detect an increase in neutralization breadth, consistent with the majority of vaccine studies to date. Linear epitope mapping results also showed a lack of substantial increase in linear epitope binding response following IDLV-1176 immunization. One caveat for linear epitope mapping analysis is that 1086.C Env strain is included in the array mapping library, whereas 1176.C strain is not included. However, binding response following IDLV-1086 boost was cross-clade for multiple epitopes including strong V3 linear epitope binding response. Enhanced binding to consensus sequences and other clade C sequences is still expected if there had been a substantial boost of binding response by 1176.C immunization. We also observed that although plasma antibody titers against 1086.C and 1176.C gp140 Envs increased following the heterologous boost at week 109, they did not reach the same levels observed following the autologous boost at week 53. The majority of memory B cells isolated before (week 57) and after the heterologous boost (week 109) cross-reacted with both envelopes, suggesting that the immunization with IDLV-1176.C did not effectively engage memory B cells reacting only with the 1176.C envelope. This phenomenon, described as original antigenic sin, where boosting with an immunogen different from the priming immunogen results in boosting of the antibody response to the priming immunogen while interfering with production of antibody to the boosting immunogen, has been previously described for several pathogens^15^, including HIV and Influenza^35,36^. In the setting of HIV vaccination it has been shown that the sequence of administration of HIV Env immunogens is important for determining the breadth of anti-Env antibody responses, and can be used to overcome this phenomenon^37^. Recent studies suggest that immunization with sequentially evolved HIV-1 envelopes may be required to induce neutralization breadth^14,38^. Our results showing that IDLV immunogens can be given repeatedly and result in boosting despite the development of short-lived anti-vector immunity suggest that the IDLV platform could be used to elicit durable responses if the right sequence of immunogens can be identified. The ability of IDLV expressing sequentially evolved HIV-1 envelopes to induce bnAbs is currently being evaluated.

To determine whether the durable immune responses observed in the vaccinated animals were associated with the persistence of vector at the injection sites, we tested for vector DNA and RNA in muscle biopsies taken post immunization. IDLV DNA was detected in all the vaccinated macaques up to 6 months post immunization, and IDLV RNA was detected in mouse muscle tissue up to 3 months post immunization. Our results are in line with previously reported data demonstrating efficient and stable transgene expression by IDLV in murine muscle cells^23^.

IDLV technology presents a safer alternative to integrase-competent lentiviral vectors for the expression of transgenes. However, despite two mutations abrogating integrase function, low levels of illegitimate integration have been observed^22–26^^,^. A significant number of these illegitimate integration events occur at sites of chromosomal breakage and are mediated by non-homologous end-joining mechanisms^26,39^. In the present study, we detected illegitimate integration in the muscle biopsy taken 1 month after the last injection with IDLV-1176 in one of six animals. In a second biopsy taken from the same animal at a later time point (6 months post injection), we could not detect any integration events, despite the presence of vector DNA. This supports the stochastic nature of the detected integration event. Additional modifications of IDLV, such as deletion of the 3’ polypurine tract to promote preferential formation of 1-LTR circular episomes^40^, may limit the linear form of vector DNA that can integrate much more efficiently than supercoiled DNA^41,42^, thereby enhancing the IDLV safety profile. In summary, our data demonstrate that IDLV-Env immunization can induce robust and durable immune responses, making it an attractive vector for HIV-1 candidate vaccines, especially in conjunction with optimized HIV-1 envelope glycoprotein immunogens for induction of bnAbs and other functional immune responses.

## Methods

### Construction of IDLV-Env plasmid

The clade C HIV-1 Env C.1176 gp140 glycoprotein^18^ was cloned into a simian immunodeficiency virus (SIV)-based self-inactivating lentiviral transfer vector^43^ downstream of the internal cytomegalovirus (CMV) promoter (pGAE-CMV-C.1176gp140Env-Wpre). The transfer vector pGAE-CMV-GFP-Wpre expressing the GFP, the integrase (IN)-defective packaging plasmid pAd-SIV-D64V containing the D64V amino acid mutation in the integrase catalytic triad to abolish the IN activity, and the vesicular stomatitis virus envelope G protein (VSV-G) pseudotyping vectors from Indiana or New Jersey serotypes (pVSV.GIND and pVSV.GNJ) have been previously described^44,45^ (accession numbers: AJ318514.1; V01214.1).

### Vector production and validation

As previously described in Negri et al.^5^, human epithelium kidney 293T Lenti-X cells (Clontech Laboratories, Mountain View, CA) were maintained in Dulbecco’s modified Eagle's medium (Thermo Fisher Scientific, Waltham, MA) supplemented with 10% fetal bovine serum (GE Healthcare Life Sciences, HyClone Laboratories, South Logan, UT) and 100 units per mL of penicillin–streptomycin–glutamine (Thermo Fisher Scientific). For production of recombinant IDLV, 3.5 × 10^6^ Lenti-X cells were seeded on 100 mm diameter Petri dishes and transfected with 12 µg per plate of a plasmid mixture containing transfer vector, packaging plasmid, and VSV.G plasmid in a 6:4:2 ratio, using the JetPrime transfection kit (Polyplus Transfection Illkirch, France) following the manufacture’s recommendations. At 48 and 72 h post transfection, culture supernatants were cleared from cellular debris by low-speed centrifugation and passed through a 0.45 μm pore size filter unit (Millipore, Billerica, MA). Filtered supernatants were concentrated by ultracentrifugation for 2 h at 23,000 RPM on a 20% sucrose cushion. Pelleted vector particles were resuspended in 1× phosphate-buffered saline (PBS) and stored at −80 °C until further use. Each IDLV-Env stock was titered using a reverse transcriptase (RT) activity assay (RetroSys RT ELISA kit, Innovagen, Lund, Sweden) and the corresponding transducing units (TUs) calculated by comparing the RT activity of each IDLV-Env stock to the RT activity of IDLV-GFP stocks with known infectious titers^6,45^. HIV-Envelope expression was confirmed on 293T cells transduced with IDLV-Env on both supernatants and cell pellets by gp120 ELISA (HIV-1 gp120 Antigen capture assay; Advanced Bioscience Laboratories, Rockville, MD).

### Animals and immunization protocol

The six Indian origin rhesus macaques (*Macaca mulatta*) used in this study were housed at BIOQUAL, Inc. in accordance with the recommendations of the Association for Assessment and Accreditation of Laboratory Animal Care International Standards and with the recommendations in the Guide for the Care and Use of Laboratory Animals of the United States–National Institutes of Health. The Institutional Animal Use and Care Committee of BIOQUAL approved these experiments (study #15–029). When immobilization was necessary, the animals were sedated by intramuscular injection with 10 mg kg^-1^ of ketamine HCl. All efforts were made to minimize suffering. Details of animal welfare and steps taken to ameliorate suffering were in accordance with the recommendations of the Weatherall report, “The use of non-human primates in research” (https://acmedsci.ac.uk/policy/policy-projects/use-of-non-human-primates-in-research). Animals were housed in climate controlled facility with an ambient temperature of 21–25 °C, a relative humidity of 40–60%, and a 12 h light/dark cycle. Animals were socially housed when possible or individually housed if no compatible pairing could be found. Animals also received appropriate environmental enrichment. The animals were housed in suspended stainless steel wire-bottomed cages and provided with a commercial primate diet and fresh fruit twice daily, with water freely available at all times. Animals were immunized intramuscularly with IDLV-Env with 3 × 10^8^ TUs per animal per immunization in 2 mL injection volume divided into two sites (left and right thighs). Peripheral blood was obtained prior to immunization, 2 weeks after immunization, and at monthly intervals throughout the study. The tattoos to identify the injection sites on the quadriceps muscles were performed using the scream ink from worldwide tattoo supply (City Of Industry, CA, USA). Muscle biopsies were performed using 5 mm disposable biopsy punches (Sklar instruments, West Chester, PA, USA) and snap frozen in liquid nitrogen.

### Mice immunization

The 6–8-week-old BALB/c female mice (Harlan Italy, S. Pietro al Natisone, Italy) were housed in accordance with the European Union guidelines and Italian legislation and were immunized once intramuscularly with 2.7 × 10^7^ RT units of IDLV-GFP or with an empty vector (IDLV-Empty) in a final volume of 0.2 mL^46^. At 3 months post immunization, mice were euthanized and thigh muscles were collected.

### Direct ELISAs

High binding EIA/RIA 384 well plates (Corning) were coated overnight with 2 µg mL^−1^ of either C.1086 gp140 or Ce.1176 gp140 proteins in coating buffer (KPL, Gaithersburg, MD). After 1 wash with washing buffer (1× PBS+0.1% Tween-20) plates were treated for 1 h at room temperature with 40 μL per well of blocking buffer (PBS containing 4% (wt/vol) whey protein–15% normal goat serum–0.5% Tween-20). Serial threefold dilutions of plasma (from 1:3000 to 1:729,000) or monoclonal antibodies (from 100 μg mL^−1^ to 0.5 ng mL^−1^) in blocking buffer were added to the plates (10 μL per well) in duplicate and incubated for 1.5 h at room temperature. The Rhesus B12 IgG (b12R1) was used to develop standard curves (range 100 to 0.005 ng mL^−1^, with each dilution assayed in duplicate). Abs were detected by adding 10 μL per well of horse radish peroxidase (HRP)-conjugated, polyclonal goat anti-monkey IgG (Rockland, Gilbertsville, PA) diluted in blocking buffer (1:6000) and by adding 20 μL per well the SureBlue Reserve TMB microwell peroxidase substrate and stop solution (KPL, Gaithersburg,MD). Binding titers were analyzed as area under curve of the log transformed concentrations (Softmax Pro 7, Molecular Devices LLC, CA). For memory B-cell culture supernatants, direct ELISAs were performed as described above with the following modifications: plates were coated with 2 µg mL^−1^ of gp140 Env proteins or 0.5 µg mL^−1^ of polyvalent goat anti-human Ig Ab (Life Technologies, Cat# H17000) to measure IgG, IgA, and IgM levels, diluted in 0.1 M NaHCO_3_ solution. Culture supernatants were tested in a 1:3 dilution in blocking buffer. The 19B mAb was used to develop standard curves. After 2 washes with washing solution, secondary HRP-conjugated antibodies (Jackson ImmunoResearch, Cat. no. 109-035-098, 109-035-129, and 109-035-011) were added at lot-specific optimal concentrations for 1 h. After 4 washes, plates were developed for 10 min using 15 µL per well SureBlue Reserve TMB microwell peroxidase substrate before adding 15 µL per well of 0.1 M HCl. All of the following criteria had to be met to define culture supernatant positivity for binding to Env immunogens: measurable IgG, IgA, or IgM levels; OD_450_ > 0.1 and > 2 × OD_450_ reads from blank wells; OD_450_ > 120% OD_650_.

### Anti-VSV-G neutralizing Ab assay

Neutralization of VSV-G-pseudotyped GFP-expressing lentiviral vectors (LV-GFP) was measured in 24-well culture plates using GFP gene expression to quantify reductions in LV transduction in 293T cells. Serum samples were assayed at twofold dilutions starting at 1:500. Neutralization titers (50% inhibitory dose (ID_50_)) are expressed as the highest dilution of serum that results in 50% reduction in fluorescence when compared to vector treated with week 0 sera.

### IgG purification and concentration

IgG was isolated from plasma using protein G columns as described in Nelson et al.^47^ (protein G resin prepacked into 96-well depletion plates (GE Healthcare)). Plasma was diluted twofold with Tris-buffered saline (TBS) (pH 7.5), and 200 µL of the diluted sample was added per well. The plates were incubated at room temperature, with shaking, for 1 h. The unbound fractions were removed by centrifugation at 700 × *g* for 3 min. Wells were then washed 3 times with 400 µL of TBS to remove loosely bound material. The IgG bound to the resin was eluted with 200 µL of 2.5% glacial acetic acid (pH 2.51) and immediately neutralized with 120 µL of 1 M Tris-HCl (pH 9.0). The eluted IgG fractions were concentrated using Amicon Ultra centrifugal filters (Millipore) with a 30,000 molecular-weight cutoff. The sample volume was reduced to 50 µL by centrifugation at 14,000 × *g* in a microcentrifuge precooled to 4 °C. A buffer exchange was then performed using 2.0 volumes of PBS, pH 7.5. The concentrated IgG was assayed for protein concentration using a NanoDrop 8000 spectrophotometer (Thermo Fisher Scientific) using the IgG reference setting and then diluted to 1 mg mL−^1^ with PBS.

### Surface plasmon resonance

To assess the reactivity to gp120 and gp140 of serum purified IgG, SPR binding assays were performed on a Biacore 4000 (GE Healthcare) maintained at 25 °C. Purified IgG samples from immunized animals at each time point between 0 and 133 weeks post immunization were tested for binding to HIV-1 Env antigens that included 1086.C gp140C and 1086.C gp120. Env protein antigens were immobilized using standard amine coupling chemistry and biotinylated peptides were captured on streptavidin-coated sensors^48,49^. Each antigen was immobilized in duplicate spots on the sensor chip and IgG samples at 100 μg mL^−1^ were injected over each of the antigen surfaces and binding responses following subtraction of background (week 0 samples) monitored for post injection response and dissociation rate (*k*_d_) measurements. Avidity score, binding responses (RU), and dissociation rates (*k*_d_) were calculated as described earlier^50^.

### HIV neutralization assays

Neutralization of Env-pseudotyped viruses was measured in 96-well culture plates using Tat-regulated firefly luciferase (Luc) reporter gene expression to quantify reductions in virus infection in TZM-bl cells^51,52^. A panel of 12 viruses, representative of the global HIV-1 strains neutralization panel^18^, was used to measure serum neutralization: SVA-MLV (negative control for non-specific activity in the assay), HIV MW965 (tier 1), HIV Ce1086_B2 (autologous, tier 2), and HIV Ce1176_A3 (autologous, tier 2), 25710-2.43 (Clade C Tier 2), TRO.11 (Clade B Tier 2), BJOX002000.03.2 CRF07_ BC (Tier 2), X1632-S2-B10 (Clade G Tier 2), 246-F3_C10_2 (Clade AC Tier 2), CH119.10 CRF07_BC (Tier 2), Ce703010217_B6 (Clade C Tier 2), CNE55 CRF01_AE (Tier 2). A panel of five viruses was used to measure mAbs neutralization: SVA-MLV (negative control for non-specific activity in the assay), HIV MW965 (tier 1), HIV Ce1086_B2 (autologous, tier 2), and HIV Ce1176_A3 (autologous, tier 2), 25710-2.43 (Clade C Tier 2). Heat-inactivated (56 °C, 1 h) serum samples were assayed at threefold dilutions starting at 1:20. The mAbs were assayed at threefold dilutions starting at 1:18.5 or 1:50. Neutralization titers (ID_50_) are the serum dilutions at which relative luminescence units (RLUs) were reduced by 50% compared to RLUs in virus control wells after subtraction of background RLUs in cell control wells. A response was considered positive if the post-immunization ID_50_ was 3 times higher than the pre-immune ID_50_ and 3 times greater than the signal against the murine leukemia virus (MLV)-pseudotyped negative control virus.

### Memory B-cell phenotyping and sorting

Macaque memory B cells were stained with both AlexaFluor 647 (AF647) and Brilliant Violet (BV421)-tagged HIV-1 1086.C gp140 and 1176.C gp140 and sorted using a BD FACSAria II (BD Biosciences, San Jose, CA). The flow cytometry data were analyzed using FlowJo (Treestar, Ashland, OR) as previously described^53^. Briefly, the following Abs were used for memory B-cell phenotyping and sort: CD20 FITC (BD Biosciences, catalog no. 347673, clone L27); IgD PE (Southern Biotech, catalog no. 2030-09); CD16 PE-Cy7 (BD Biosciences, catalog no. 557744, clone 3G8); CD27 APC-Cy7 (BioLegend, clone O323); CD14 BV570 (BioLegend, catalog no. 301832, clone M5E2); and CD3 PerCP-Cy5.5 (BD Biosciences, catalog no. 552852, clone SP34-2). All antibodies were titered in advance and used to stain macaque PBMCs at optimal concentrations for flow cytometry. The gating strategy used for B-cell phenotyping and sorting is shown in Supplementary Figure 3.

### Memory B-cell culture

To induce proliferation and differentiation of Rhesus memory B cells, we used a previously optimized method derived from our human memory B-cell culture system^12^. Memory B cells specific for either 1086.C or 1176.C were flow sorted as described above in bulk into wells containing 5000 MS40L feeder cells, RPMI-1640 supplemented with 15% FBS, 1 mM sodium pyruvate, 1% non-essential amino acids, 25 mM HEPES buffer, 2.5 μg mL^−1^ ODN2006 (Invivogen, Cat. no. TLRL-2006-5), 5 μM CHK2-inhibitor (Calbiochem, Cat. no. 220486-1MG), 100 ng mL^−1^ recombinant human interleukin (IL)-21 (Peprotech, Cat. no. 2001-21), 10 ng mL^−1^ recombinant Human BAFF (Peprotech, Cat. no. 310-13), 200 U mL^−1^ IL-2 (from the myeloma IL-2 producing cell line IL2-t6, kindly provided by Dr. Antonio Lanzavecchia, IRB, Bellinzona, Switzerland), and 100 μL supernatant of the Herpesvirus papio (HVP)-infected Baboon cell line S594 (NHP Reagent Resource). The concentration of each supplement was previously determined to achieve optimal in vitro stimulation. Following overnight incubation at 37 °C in 5% CO_2_, memory B cells were diluted to a concentration of 1 cell per well and cultured for 2 weeks in round bottom tissue culture plates containing 5000 non-irradiated MS40L feeder cells. Culture medium was refreshed 7 days after plating and harvested for binding against 1086.C gp140 and 1176.C gp140 7 days later (2 weeks post plating).

### Isolation and expression of heavy and light chain genes

Heavy (IGHV) and light (IGKV, IGLV) chain genes were isolated via single-cell PCR on flow-sorted memory B cell^54,55^. Antigen-specific flow sorting was performed using fluorophore-labeled HIV-1 Env 1086.C gp140, HIV-1 Env 1086.C gp120 V1/V2 tag, and RSC3/RSC3Δ371 (resurfaced stabilized core 3 probe that preferentially bind to CD4bs antibodies)^56^ recombinant proteins. Plasmids encoding the IGHV, IGKV, and IGLV genes were generated and used for rmAb production by transient transfection of 293F cells (Life Technologies, Grand Island, NY). The paired Ig heavy and light chains were co-transfected (1 μg of each) into 80–90% confluent 293F cells using PolyFect (Qiagen, Valencia, CA) following the manufacturer’s instructions. Fresh culture medium, supplemented with 2% FBS, was added to 293F cells 6 to 8 h after transfection^54^. The supernatants were collected 72 h post transfection and screened for reactivity against the following HIV Env proteins by small-scale ELISA (see Direct ELISAs section above for assay details): MN gp41, C.1086 gp140, C.1086 gp120, C.1086 V1V2 Tags, C. 1086 V2 Tags, C.1086 V1V2 N156Q, CH0505 TF gp120, CH0505 TF gp120 d371, YU2 gp120, YU2 D368R, C.Con V3 loop, AE.A244 gp120, AE.A244 gp120 V1V2 Tags, B63521 gp120, B63521 gp120 V1V2 Tags, Con C gp120, and M Con S gp140. Fourteen mAbs were selected for large-scale production based upon their binding profile. Heavy and light chains for the 14 selected mAbs were cloned into the pcDNA3.1+ mammalian expression vector. Plasmids were transiently transfected into Expi 293i cells (Invitrogen; catalog no. A14527) using the ExpiFectamine (Life Technologies, Gibco, Cat. no. A14524) transfection kit following the manufacturer’s instructions. The produced antibodies were purified using protein A beads (BioVision, Inc., Cat. no. PI-20334) as described previously^54^.

### ADCC

The ADCC activity of mAbs against CEM.NKR_CCR5_ infected with C.1086 or MW965.25 infectious molecular clones (IMCs) was assessed as previously described^57^. Briefly, CEM.NKRCCR5 cells (NIH AIDS Reagent Program, Division of AIDS, NIAID, NIH; from Alexandra Trkola)^58^ were infected with a C.1086 or MW965.25 IMCs that encode the *Renilla* luciferase reporter gene and preserves all HIV-1 open reading frames using dextran-diethylaminoethyl (DEAE). Cryopreserved human PBMCs from an HIV-seronegative donor with the heterozygous 158F/F genotype for Fc-gamma receptor IIIa were used as the source of the effector cells. The infected CEM.NKR_CCR5_ cells and the effector cells were incubated with serial fivefold dilutions of mAbs starting at 50 µg mL^−1^ in R10 medium for 6 h at 37 °C. Killing activity was measured as reduction in RLUs compared to RLUs in virus control wells. Results were considered positive if the percent specific killing was greater than 15%.

### Heavy and light chain genes sequence analysis

DNA sequence base calling was performed using Phred^59^ and contigs assembled using an in-house bioinformatics pipeline. The heavy (IGHV) and light (IGKV, IGLV) chain gene sequences of isolated mAbs were computationally analyzed and immunogenetics (gene segments, mutation frequency, CDR3 length) determined using the Cloanalyst program^17,59,60^. Immunogenetic information was assigned using the 2017 Cloanalyst Macaca mulatta gene segment library^61^. Antibody sequences were partitioned into clones with Cloanalyst using a test for clonal relatedness with the following criteria: identical or highly similar heavy and light chain V and J gene segment assignments, identical CDR3 lengths, and a CDR3 sequence consistency measure based on an adapative CDR3 sequence similarity cutoff that is inversely proportional to V gene mutation frequency^17^.

### Linear epitope mapping

Serum epitope mapping of heterologous strains was performed as previously described^62,63^ with minor modifications. Briefly, a peptide library of overlapping peptides (15-mers overlapping by 12), covering 7 full-length HIV-1 gp160 Env consensus sequences (clades A, B, C, and D, group M, CRF1, and CRF2) and 6 vaccine and laboratory strain gp120 sequences (A244_AE, TH023_AE, MN_B, 1086_C, TV1_C, and ZM651_C), was printed onto epoxy glass slides (JPT Peptide Technologies GmbH, Germany). Microarray binding was performed using the HS4800 Pro Hybridization Station (Tecan, Männedorf, Switzerland). All arrays were blocked with blocking buffer (PBS+1% milk+5% normal goat serum+0.05% Tween-20) for 1 h at 30 °C, followed by a 2 h of incubation at 30 °C with sera diluted 1:50 in blocking buffer. Arrays were incubated for 45 min at 30 °C with goat anti-Hu IgG conjugated with DyLight649 (Jackson ImmunoResearch, PA) (1.5 µg mL^−1^ final concentration) diluted with blocking buffer. Washes between all steps were with PBS containing 0.1% Tween-20. Arrays were scanned at a wavelength of 635 nm using an Axon Genepix 4300 Scanner (Molecular Devices, Sunnyvale, CA, USA) at a PMT setting of 580, 100% laser power. Images were analyzed using Genepix Pro 7 software (Molecular Devices). Binding intensity of the post immunization serum to each peptide was corrected with its own background value, which was defined as the median signal intensity of the prebleed serum for that peptide plus 3 times the standard errors among the three subarray replicates present on each slide.

### Vector detection in muscle biopsies

DNA and RNA were isolated from muscle after tissue homogenization using the SV Total RNA isolation system (Promega) following the manufacturer’ instructions for isolation of both DNA and RNA. Standard curves for total and integrated IDLV DNA quantification in monkey’s muscle biopsies were generated using serial dilutions of genomic DNA extracted from CMMT macaca mulatta cells (ATCC CRL-6299) stably transduced with a neomycin resistant integrase-competent lentiviral vector-expressing GFP (CMMT-LV-Neo). The reference gene β-actin was used as loading control. 50 ng of RNA extracted from mice thigh muscles were reverse transcribed with random examer using the ImProm-II™ Reverse Transcription System kit (Promega) following the manufacturer instructions. Complementary DNA was amplified using GFP- and β-actin-specific primers with AmpliTaq Gold™ 360 Master Mix (Thermo Fisher Scientific). The C26 murine cell line constitutively expressing GFP was used as a positive control (C26/GFP). RT minus reactions were performed to exclude the presence of contaminating vector DNA. PCR reactions were performed using the primer sets and conditions showed in Supplementary Table 3.

### Statistical analysis

Comparisons between time points were made using the Wilcoxon signed-rank test because of the small sample size. All computations were made using SAS v9.4 (SAS Institute, Inc.).

## Electronic supplementary material

Supplementary Information

Description of Supplementary Data

Supplementary Data 1

Supplementary Data 2

## Data Availability

The data that support the findings of this study have been deposited to figshare^64^ at https://figshare.com/articles/Blasi_et_al_Comm_Biol_2018_Data_pzfx/6849011. Plasmid sequences can be found in Supplementary Data 2.
