# Supplementary material for: IDLV-HIV-1 Env vaccination in non-human primates induces affinity maturation of antigen-specific memory B cells
Source: Commun Biol. 2018 Sep 5;1:134. doi: 10.1038/s42003-018-0131-6 (PMC6125466; doi:10.1038/s42003-018-0131-6)
Supplement: Supplementary file 1 — Supplementary Information [file 42003_2018_131_MOESM1_ESM.pdf]

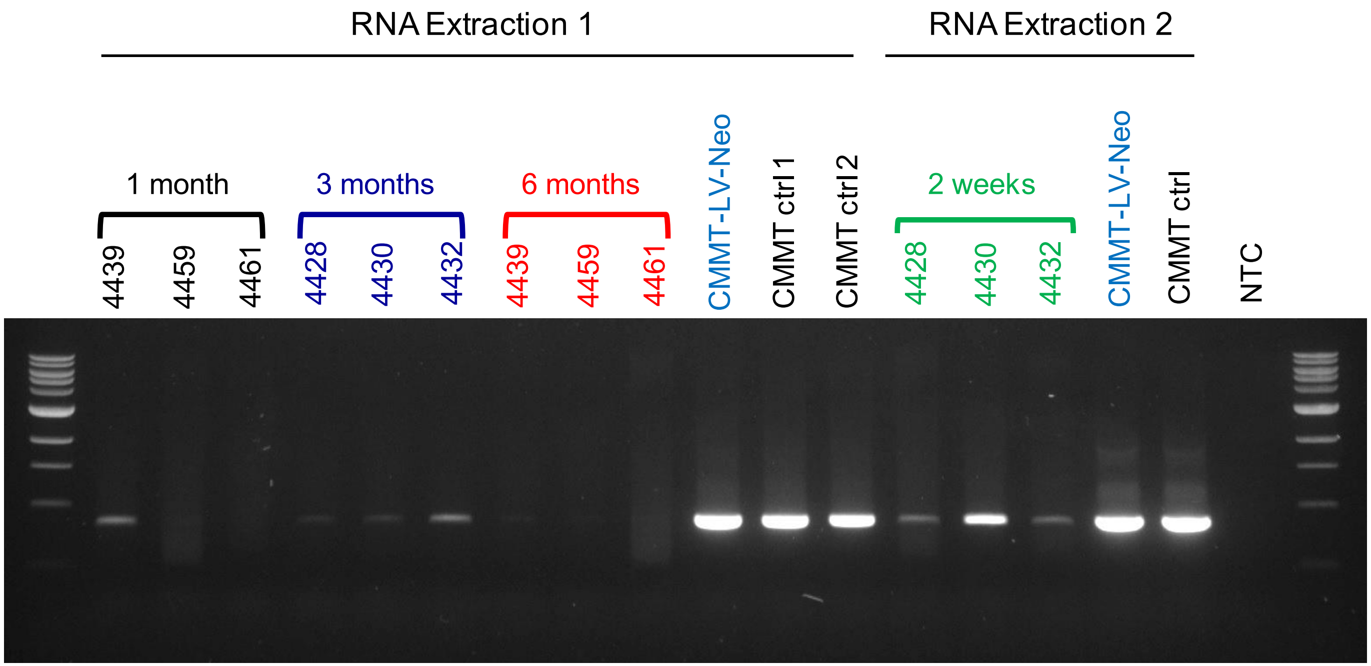

Supplementary Figure 1. β−actin PCR on retro−transcribed RNA extracted from macaque muscle biopsies at the indicated time points post IDLV−injection. Genomic DNA extracted from the negative (CMMT) and the positive control (CMMT-LV-Neo) corresponding to ~$1.2x10^4$ cells were also amplified. One set of negative and positive controls is shown for each RNA extraction.

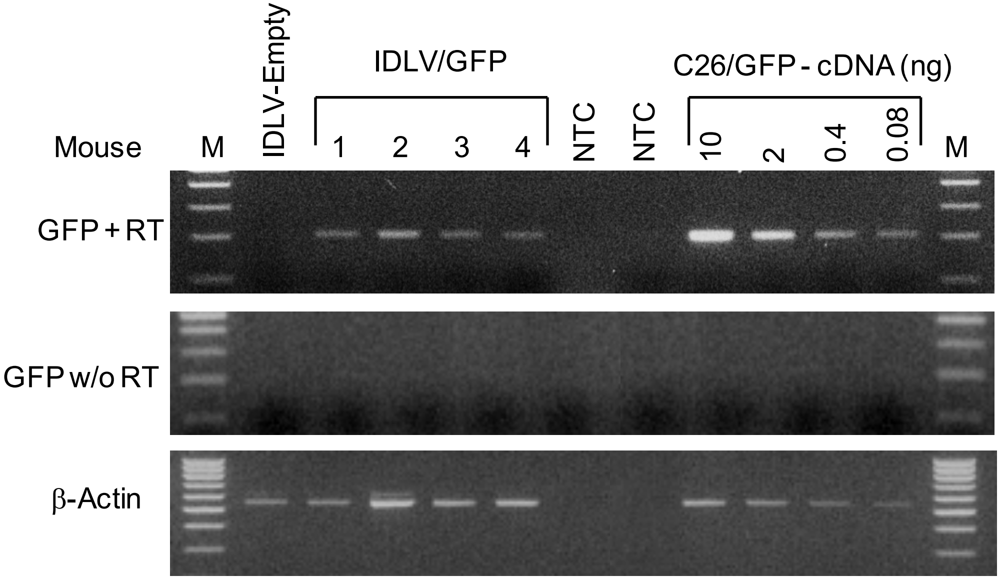

Supplementary Figure 2. IDLV-RNA persists at the site of injection at 3 months post-immunization. RT-PCR for GFP or **β**-Actin on RNA extracted from mouse thigh muscles at 3 months from IDLV/GFP injection.

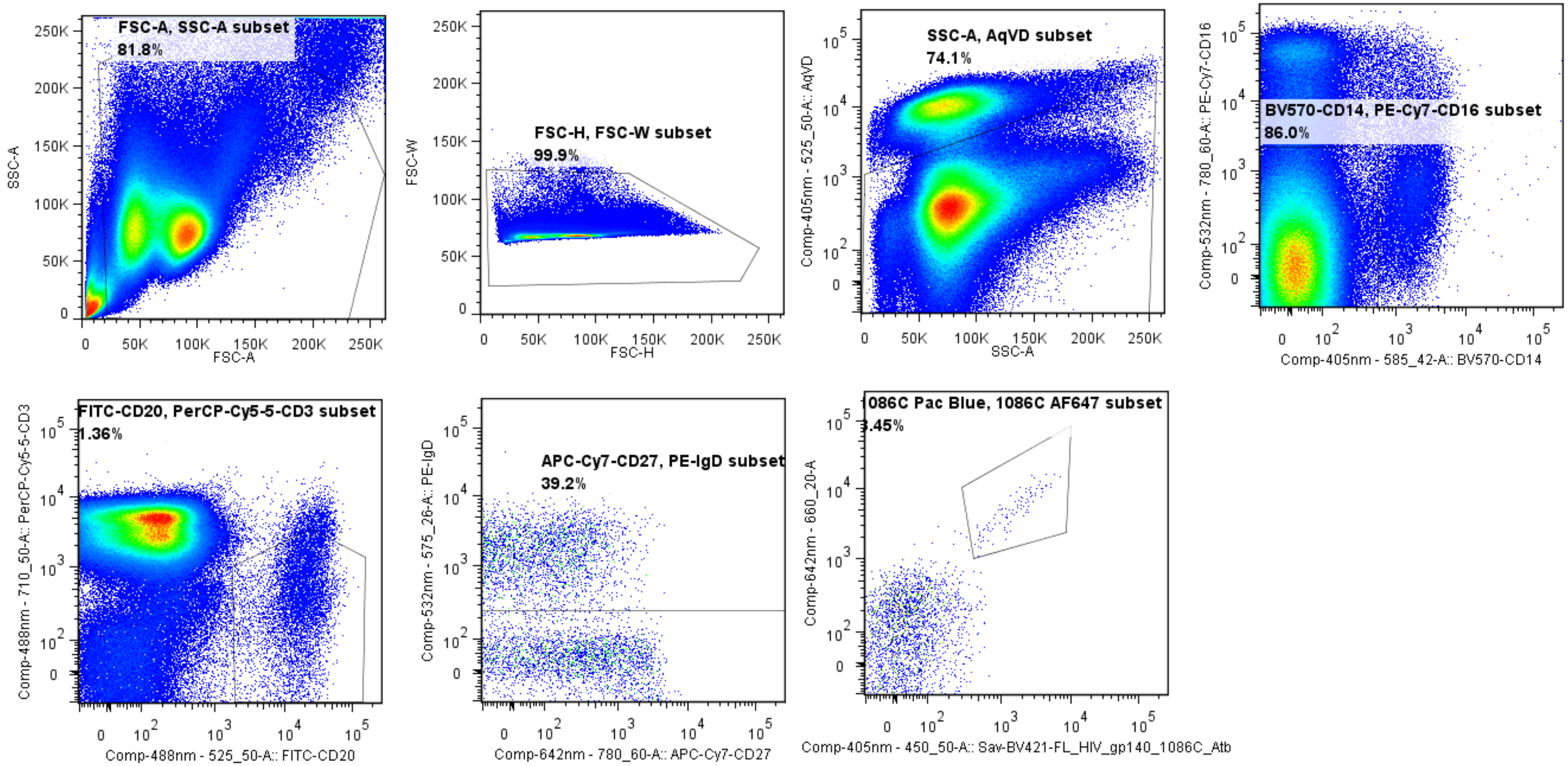

Supplementary Figure 3. Antigen specific memory B cell sort gating strategy. Memory B cells were sorted as IgD-/CD27all

Supplementary Table 1. Genetic characteristics of clonally related mAbs.

	Clone number	Ab ID	VH Gene	HCDR3 Length (aa)	% Mut	Light chain	VL Gene	LCDR3 Length (aa)
V2 mAbs clones	1	910895	IGHV3-ah*02	14	3.5	λ	IGLV3-i*01	11
		912813	IGHV3-ah*02	14	8	λ	IGLV3-i*01	11
		913338	IGHV3-ah*02	14	6.6	λ	IGLV3-i*01	11
	2	911883	IGHV4-g*01	17	9.7	λ	IGLV3-k*02	11
		912818	IGHV4-g*01	17	9.7	λ	IGLV3-k*02	11
		912819	IGHV4-g*02	17	8.7	λ	IGLV3-k*02	11
	3	911884	IGHV4-f*02	17	9.4	λ	IGLV3-k*02	11
		912805	IGHV4-f*02	17	9.4	λ	IGLV3-k*02	11
	4	912809	IGHV3-ah*01	15	10.8	λ	IGLV3-k*02	11
		912810	IGHV3-ah*01	15	10.8	λ	IGLV3-k*02	11
	5	912814	IGHV4-f*02	13	7.6	κ	IGKV2-x*01	9
		912815	IGHV4-f*02	13	8	κ	IGKV2-x*01	9
CD4bs mAbs clone	6	913332	IGHV4-n*03	20	8.2	λ	IGLV2-b*06	10
		913344	IGHV4-n*03	20	8.2	λ	IGLV2-b*06	10
		913345	IGHV4-n*03	20	7.9	λ	IGLV2-b*06	10
V3 mAbs clone	7	910896	IGHV4-e*01	18	5.9	κ	IGKV1-x*01	9
		910898	IGHV4-e*01	18	9	κ	IGKV1-x*01	9

MW965.26 Neutralization						
	Animal ID					
Week	4428	4430	4432	4439	4459	4461
0	<20	<20	<20	<20	<20	<20
96	28	1036	33	95	96	187
109	67	3492	55	102	164	315
111	31	13706	48	64	167	256
113	26	984	49	39	259	265

ID50
<20
20-100
100-1050
1050-10,000
>10,000

Supplementary Table 2. Serum neutralization activity against the clade C tier1 virus MW965.26. Values are the serum dilutions at which relative luminescence units (RLU) were reduced by 50% compared to RLU in virus control wells after subtraction of background RLU in cell control wells. A response was considered positive if the post-immunization ID50 was 3 times higher than the pre-immune ID50 and 3 times greater than the signal against the MLV-pseudotyped negative control virus. No neutralization against the tier 2 viruses representative of the global neutralization panel (1086.C_CladeC, 1176.C_Clade C, 25710-2.43_Clade C, TRO.11_Clade B, BJOX002000.03.2 CRF07_BC, X1632-S2-B10 Clade G, 246-F3_C10_2 Clade AC, CH119.10 CRF07_BC, Ce703010217_B6 Clade C, CNE55 CRF01_AE) was detected.

Supplementary Table 3. Primers and PCR conditions for the detection of SIV/IDLV.

Target	Primer	Sequence (5'-3')	PCR conditions
**Total SIV-IDLV DNA**	CMVfor<br>CMVas	ACG CCA ATA GGG ACT TTC CAT TGA C<br>ACG CCC ATT GAT GTA CTG CCA AA	95°C 30s - 60°C 30s - 72°C 30s for 35 cycles
**Integrated SIV-IDLV DNA**			
Outer	CMVfor<br>Alu1<br>*Alu2*	ACG CCA ATA GGG ACT TTC CAT TGA C<br>TCC CAG CTA CTG GGG GAG GCT GAG G<br>GCC TCC CAA AGT GCT GGG ATT ACA G	95°C 30s - 60°C 30s -72°C 3min for 18 cycles
Nested<br>(these primers also for RNA)	EnvFor<br>EnvRev	CTA CGA GAA GGA GGT GCA CAA<br>TCA GCT TCA CGC AGG GCT TCA	95°C 30s - 60°C 30s - 72°C 30s for 25 cycles
Nested<br>(these primers also for RNA)	EnvFor1<br>EnvRev1	GCT GTA CAA GTA CAA GGT GGT<br>GTT GGA CTG CTG CTG CAC GA	95°C 30s - 60°C 30s - 72°C 30s for 25 cycles
**β-Actin**	Actin F<br>Actin R	ATC TGG CAC CAC AAC AAT GAG CTG CG<br>CGT CAT ACT CCT GGA TCC ACA TCT GC	94°C 30s - 55°C 30s - 72°C 30s for 30 cycles
**GFP**	GFPfor2<br>GFPrev2	GCT ACC CCG ACC ACA TGA AGC AGC A<br>TGT GCC CCA GGA TGT TGC CG	94°C 30s - 60°C 30s - 72°C 30s for 35 cycles
