# Supplementary material for: IDLV-HIV-1 Env vaccination in non-human primates induces affinity maturation of antigen-specific memory B cells
Source: Commun Biol. 2018 Sep 5;1:134. doi: 10.1038/s42003-018-0131-6 (PMC6125466; doi:10.1038/s42003-018-0131-6)
Supplement: Supplementary file 2 — Description of Supplementary Data [file 42003_2018_131_MOESM2_ESM.docx]

**Supplementary Data 1**

*a* An OD of 2 times that for mock-transfected wells was considered positive and is highlighted by shading. DP: double positive; diff: differential binders. The sort strategy used to isolate each of the listed mAbs, the heavy and light chains gene usage and mAbs reactivity against a panel of HIV-1 envelope proteins are shown. gp120 specific mAbs defined as antibodies that bind to the 1086.C gp140 and gp120 proteins but not to MNgp41; gp41 specific mAbs defined as antibody that bind to gp140 and MNgp41 proteins, but not to gp120. The bolded mAbs were selected for large scale production and functional screenings (ADCC and Neutralization).

**Supplementary Data 2**

Sequences of transfer vector plasmid (pGAE-CMV-C.1176gp140Env-Wpre), envelope plasmid (pCAGG-NJ-G) and packaging plasmid (pAd-SIV-D64V) used to generate IDLV particles.
