# Supplementary material for: IDLV-HIV-1 Env vaccination in non-human primates induces affinity maturation of antigen-specific memory B cells
Source: Commun Biol. 2018 Sep 5;1:134. doi: 10.1038/s42003-018-0131-6 (PMC6125466; doi:10.1038/s42003-018-0131-6)
Supplement: Supplementary file 3 — Supplementary Data 1 [file 42003_2018_131_MOESM3_ESM.pdf]

Supplementary Data 1. Genetic characteristics and Env specificity of mAbs produced in small scale from Env-specific B cells isolated from monkey 4430

	Heavy Chain					Light Chain			Small-scale transfection ELISA (OD)[a]								
Sort Strategy	Ab ID	VH Gene	HCDR3 Length (aa)	% Mut	Light chain	VL Gene	LCDR3 Length (aa)	IgG Quant (ug/ml)	MN gp41	C.1086 gp140	C.1086 gp120	C.1086 V1V2 Tags	CH0505 TFgp120	CH505 TF gp120 d371	YU2 gp120	YU2 D368R	C.Con V3 loop
1086.C gp140 DP	**910893**	IGHV3-ah*02	12	10.4	λ	IGLV3-k*02	11	0.95	0.00	3.35	3.44	3.48	0.00	0.00	0.00	0.01	0.00
1086.C gp140 DP	**910894**	IGHV4-j*02	20	9.3	λ	IGLV2-d*01	10	3.28	0.01	3.11	3.32	0.00	1.02	0.00	3.19	2.98	0.01
1086.C gp140 DP	**910895**	IGHV3-ah*02	14	3.5	λ	IGLV3-i*01	11	0.07	-0.01	3.13	3.39	3.31	3.07	2.93	0.00	0.01	0.00
1086.C gp140 DP	910896	IGHV4-e*01	18	5.9	κ	IGKV1-x*01	9	0.01	-0.01	3.09	3.16	0.00	0.01	0.00	0.00	0.00	2.10
1086.C gp140 DP	910897	IGHV4-n*03	11	6.9	λ	IGLV3-j*05	11	0.03	-0.01	1.75	0.35	-0.01	0.06	0.00	0.03	0.05	-0.01
1086.C gp140 DP	910898	IGHV4-e*01	18	9.0	κ	IGKV1-x*01	9	1.09	0.00	3.11	3.31	0.00	3.29	3.27	0.81	1.01	3.28
1086.C gp140 DP	910899	IGHV4-j*02	10	4.9	λ	IGLV2-b*03	11	1.61	3.24	3.11	-0.01	0.00	0.00	0.00	0.00	0.00	0.00
1086.C gp140 DP	**910900**	IGHV4-g*02	13	9.0	κ	IGKV2-x*01	9	10.69	0.02	3.21	3.37	3.33	0.00	0.00	0.00	-0.01	0.00
1086.C gp140 DP	910903	IGHV4-f*02	17	13.5	κ	IGKV3-f*02	8	3.04	0.12	3.18	3.12	0.00	3.20	3.05	2.89	2.81	0.01
1086.C gp140 DP	911882	IGHV4-f*03	19	10.3	κ	IGKV1-d*02	9	0.31	0.02	3.17	3.10	0.08	0.14	0.00	2.72	2.27	-0.03
1086.C gp140 DP	**911883**	IGHV4-g*01	17	9.7	λ	IGLV3-k*02	11	2.73	0.01	3.55	3.17	3.28	1.51	2.99	0.00	0.00	-0.02
1086.C gp140 DP	**911884**	IGHV4-f*02	17	9.4	λ	IGLV3-k*02	11	1.97	0.01	3.36	3.27	3.28	0.00	0.01	0.00	0.00	-0.03
1086.C gp140 DP	911885	IGHV5-b*03	19	2.1	κ	IGKV1-c*04	9	1.00	0.04	3.26	3.11	0.06	0.02	0.00	0.00	0.00	-0.03
1086.C gp140 DP	911886	IGHV4-f*02	16	6.5	κ	IGKV2-k*03	9	1.27	0.94	3.25	0.00	0.06	0.00	0.00	0.00	0.00	-0.03
1086.C gp140 DP	911887	IGHV4-j*03	13	11.8	λ	IGLV2-d*01	10	0.59	3.25	3.28	0.00	0.07	0.00	0.00	0.00	0.00	-0.03
1086.C gp140 DP	911888	IGHV3-al*01	12	7.6	λ	IGLV8-a*01	10	0.53	0.08	3.27	3.25	0.08	2.70	2.93	1.09	0.82	-0.03
1086.C gp140 DP	**911890**	IGHV3-g*03	20	8.5	κ	IGKV1-r*01	9	3.50	0.01	3.35	3.31	0.08	3.24	3.22	3.19	2.77	-0.03
1086.C gp140 DP	911891	IGHV4-g*02	16	9.4	λ	IGLV1-d*01	11	1.56	3.40	3.32	0.00	0.09	0.00	0.00	0.01	0.00	-0.03
1086.C gp140 DP	**911892**	IGHV5-a*04	16	5.6	λ	IGLV3-j*05	11	2.39	0.02	3.45	3.41	0.10	3.43	3.45	3.34	3.40	3.31
1086.C gp140 DP	911893	IGHV3-ah*02	12	11.5	λ	IGLV8-a*01	10	1.07	0.01	3.50	3.62	0.05	3.14	3.41	1.95	1.40	-0.02
1086.C gp140 DP	911894	IGHV4-f*02	11	7.6	λ	IGLV1-h*01	11	0.90	0.03	3.28	0.00	0.07	0.00	0.00	0.00	0.00	-0.03
1086.C gp140 DP	911895	IGHV3-f*04	14	5.1	λ	IGLV5-e*01	9	5.16	0.01	3.38	3.37	0.05	3.33	3.36	2.23	1.38	3.19
1086.C gp140 DP	911896	IGHV4-n*03	13	9.3	λ	IGLV8-a*01	10	<0.001	0.01	0.48	0.00	0.06	0.00	0.00	0.00	0.00	-0.03
1086.C gp140 DP	**911897**	IGHV4-j*02	15	3.5	λ	IGLV2-j*15	11	4.79	0.30	3.42	3.41	0.07	2.49	0.00	3.18	0.00	-0.02
1086.C gp140 DP	911898	IGHV4-m*02	17	9.2	λ	IGLV2-f*01	10	3.42	0.02	3.40	3.29	0.05	2.85	3.27	0.00	0.00	-0.03
1086.C gp140 DP	**911899**	IGHV4-n*03	18	12.0	λ	IGLV1-c*06	11	3.62	0.18	3.32	3.29	0.06	3.06	0.00	0.92	0.00	-0.03
1086.C gp140 DP	**911900**	IGHV1-c*01	19	1.0	λ	IGLV2-b*06	9	5.43	0.01	3.41	3.31	0.07	0.30	0.07	1.34	1.19	-0.03
1086.C gp140 DP	**911901**	IGHV1-c*01	17	5.6	κ	IGKV2-x*01	9	5.41	0.02	3.39	3.37	0.09	3.38	3.33	0.00	0.01	3.24
1086.C gp140 DP	911902	IGHV4-f*02	20	7.2	κ	IGKV1-d*02	9	6.36	0.13	3.48	3.34	0.11	0.04	0.03	1.65	1.02	0.06
1086.C gp140 DP	911903	IGHV4-m*02	16	7.5	λ	IGLV2-j*16	11	4.76	0.02	3.23	1.77	0.09	0.00	0.00	0.00	0.00	-0.03
1086.C gp140 DP	**911904**	IGHV3-ad*02	19	0.7	λ	IGLV1-a*03	11	1.21	0.01	3.28	3.33	0.11	2.07	0.07	2.12	0.00	-0.03
1086.C gp140 DP	**911905**	IGHV2-d*01	19	1.4	κ	IGKV2-w*06	9	0.90	0.03	3.42	3.11	3.08	0.00	0.00	0.00	0.00	-0.03
1086.C gp140 DP	911906	IGHV4-g*01	13	10.8	λ	IGLV2-d*01	9	5.42	3.07	3.38	0.01	0.12	0.01	0.00	0.01	0.02	0.02

	Heavy Chain					Light Chain			Small-scale transfection ELISA (OD)											
Sort Strategy	Ab ID	VH Gene	HCDR3 Length (aa)	% Mut	Light chain	VL Gene	LCDR3 Length (aa)	IgG Quant (ug/ml)	MN gp41	C.1086 gp120	C.1086 V1V2 Tags	C.1086 V2 Tags	AE.A244 gp120	AE.A244 V1V2 Tags	B.63521 gp120	B.63521 V1V2 Tags	CH0505 TF gp120	YU2 gp120	ConC gp120 WT	M Con S gp140
1086.C gp120 V1V2 DP	912805	IGHV4-f*02	17	9.4	λ	IGLV3-k*02	11	1.93	0.01	3.54	3.48	3.84	3.42	3.84	2.86	3.43	0.00	0.03	0.06	2.84
1086.C gp120 V1V2 DP	912806	IGHV4-n*03	11	12.0	λ	IGLV3-h*01	10	2.74	0.01	3.23	3.28	3.55	3.28	3.36	3.36	3.42	3.44	3.32	3.18	3.49
1086.C gp120 V1V2 DP	912807	IGHV3-ah*01	11	6.3	λ	IGLV3-k*02	11	1.99	0.01	3.29	3.26	3.46	3.30	3.43	0.01	0.00	0.00	0.00	0.00	-0.01
1086.C gp120 V1V2 DP	912808	IGHV2-d*01	19	3.1	κ	IGKV2-w*06	9	1.07	0.02	3.17	3.32	3.56	0.00	0.05	0.02	0.00	0.00	0.00	-0.01	-0.01
1086.C gp120 V1V2 DP	912809	IGHV3-ah*01	15	10.8	λ	IGLV3-k*02	11	0.62	0.00	3.13	3.27	3.59	0.00	0.00	0.00	0.00	0.00	0.00	-0.01	-0.01
1086.C gp120 V1V2 DP	912810	IGHV3-ah*01	15	10.8	λ	IGLV3-k*02	11	0.27	0.25	2.64	2.90	2.80	-0.01	0.00	0.00	0.00	0.01	0.03	0.02	0.08
1086.C gp120 V1V2 DP	912811	IGHV1-a*07	19	13.9	κ	IGKV2-k*03	9	1.19	0.01	3.43	3.25	3.24	3.34	3.22	2.82	3.32	0.01	0.04	0.10	3.09
1086.C gp120 V1V2 DP	912813	IGHV3-ah*02	14	8.0	λ	IGLV3-i*01	11	0.16	0.00	3.17	3.18	3.36	3.27	3.37	0.03	0.00	3.04	0.00	0.00	-0.01
1086.C gp120 V1V2 DP	912814	IGHV4-f*02	13	7.6	κ	IGKV2-x*01	9	6.63	0.19	3.16	3.22	3.46	3.27	3.41	0.02	0.01	0.00	0.01	0.00	0.00
1086.C gp120 V1V2 DP	912815	IGHV4-f*02	13	8.0	κ	IGKV2-x*01	9	0.29	0.01	3.13	3.20	3.28	3.31	3.38	0.03	0.00	0.00	0.01	0.01	0.01
1086.C gp120 V1V2 DP	912816	IGHV1-a*07	19	8.7	κ	IGKV2-k*03	9	2.61	0.01	3.36	3.30	3.31	3.36	3.34	1.40	3.16	0.00	0.02	0.02	2.86
1086.C gp120 V1V2 DP	912818	IGHV4-g*01	17	9.7	λ	IGLV3-k*02	11	0.93	0.01	3.25	3.34	3.16	3.40	3.18	0.10	0.01	1.17	0.00	0.97	2.68
1086.C gp120 V1V2 DP	912819	IGHV4-g*02	17	8.7	λ	IGLV3-k*02	11	0.95	0.01	3.30	3.41	3.24	3.31	3.20	0.01	0.01	0.09	0.01	0.05	0.77
1086.C gp120 V1V2 DP	912820	IGHV3-y*01	18	14.9	λ	IGLV5-a*01	9	0.54	0.01	3.07	3.33	3.19	3.30	3.16	0.01	0.02	1.62	0.00	0.00	0.00

Sort Strategy	Heavy Chain				Light chain	Light Chain		IgG Quant (ug/ml)	Small-scale transfection ELISA (OD)							
	Ab ID	VH Gene	HCDR3 Length (aa)	% Mut		VL Gene	LCDR3 Length (aa)		MN gp41	C.1086 gp140	C.1086 gp120	C.1086 V1V2 Tags	C.1086 V1V2 N156Q	CH0505 TFgp120	CH505 TF gp120 d371	C.Con V3 loop
CH505 gp120 wt/d371 diff	913307	IGHV4-j*02	20	1.4	κ	IGKV1-g*04	9	3.90	0.01	3.30	3.25	0.00	0.00	2.85	2.48	0.00
CH505 gp120 wt/d371 diff	913308	IGHV4-m*02	19	6.5	λ	IGLV2-j*16	11	4.39	0.01	3.32	3.23	0.00	0.00	0.43	0.02	0.00
CH505 gp120 wt/d371 diff	913309	IGHV4-m*02	19	6.5	λ	IGLV2-j*16	11	<0.001	0.00	2.02	0.42	0.00	0.00	0.02	0.00	0.00
CH505 gp120 wt/d371 diff	913311	IGHV3-ai*01	15	2.1	λ	IGLV2-j*16	11	0.05	0.00	2.94	2.86	0.00	0.00	1.90	0.02	0.00
CH505 gp120 wt/d371 diff	913312	IGHV1-c*01	16	4.2	λ	IGLV2-j*16	11	4.93	0.02	3.26	3.28	0.00	0.00	2.94	2.55	0.00
CH505 gp120 wt/d371 diff	913313	IGHV3-f*04	10	4.4	κ	IGKV2-x*01	9	0.23	0.00	3.12	3.02	0.00	0.00	0.00	0.00	0.00
CH505 gp120 wt/d371 diff	913315	IGHV4-e*01	13	9.7	λ	IGLV8-a*01	11	1.22	0.01	2.88	2.99	0.00	-0.01	0.00	0.00	0.00
CH505 gp120 wt/d371 diff	913316	IGHV4-b*01	21	9.6	κ	IGKV3-a*06	8	2.41	0.02	3.58	3.43	0.00	0.00	3.30	0.76	0.00
CH505 gp120 wt/d371 diff	913317	IGHV4-j*02	18	4.5	λ	IGLV2-d*01	10	2.54	0.02	3.51	3.21	0.00	0.00	0.20	0.03	0.01
CH505 gp120 wt/d371 diff	913318	IGHV1-c*01	16	6.3	λ	IGLV3-j*05	11	3.26	0.05	3.38	3.22	0.00	0.00	0.19	0.04	0.00
CH505 gp120 wt/d371 diff	913320	IGHV4-f*02	20	5.2	λ	IGLV2-b*06	10	0.60	0.01	3.36	3.23	0.00	0.00	0.99	0.00	0.00
CH505 gp120 wt/d371 diff	913321	IGHV4-f*03	24	8.9	κ	IGKV1-d*02	9	1.31	0.02	3.39	3.31	0.00	0.00	0.60	0.00	0.01
CH505 gp120 wt/d371 diff	913322	IGHV4-f*02	20	4.9	λ	IGLV3-j*05	11	2.07	0.01	3.38	3.17	0.00	0.00	0.22	0.01	0.00
CH505 gp120 wt/d371 diff	913323	IGHV4-j*02	21	8.9	λ	IGLV2-d*01	10	1.12	0.01	3.35	3.36	0.00	0.00	0.98	0.05	0.00
CH505 gp120 wt/d371 diff	913324	IGHV4-j*02	21	8.9	λ	IGLV2-d*01	10	2.53	0.03	3.32	3.31	0.00	0.00	2.58	0.46	0.01
CH505 gp120 wt/d371 diff	913325	IGHV3-j*02	11	2.4	λ	IGLV3-h*01	9	0.12	0.00	2.72	2.96	0.00	0.00	0.12	0.32	0.00
CH505 gp120 wt/d371 diff	913326	IGHV3-j*02	11	2.4	λ	IGLV3-h*01	9	0.15	0.00	2.90	3.07	0.00	0.00	0.16	0.38	0.00
CH505 gp120 wt/d371 diff	913327	IGHV4-m*02	16	4.8	λ	IGLV2-i*01	10	0.32	0.00	1.14	1.04	0.00	0.00	0.02	0.01	0.00
CH505 gp120 wt/d371 diff	913328	IGHV3-ad*02	18	6.3	κ	IGKV2-v*01	9	1.08	0.03	3.37	3.32	0.01	0.02	3.23	3.34	3.18
CH505 gp120 wt/d371 diff	913329	IGHV4-j*02	18	3.8	λ	IGLV8-a*01	9	2.19	0.06	3.51	3.35	0.00	0.00	3.04	0.00	0.01
CH505 gp120 wt/d371 diff	913332	IGHV4-n*03	20	8.2	λ	IGLV2-b*06	10	2.01	0.02	3.39	3.30	0.00	0.00	2.59	0.01	0.00
CH505 gp120 wt/d371 diff	913333	IGHV3-x*04	18	14.2	λ	IGLV5-a*01	9	1.04	0.01	3.51	3.37	3.47	3.33	1.69	2.54	0.00
CH505 gp120 wt/d371 diff	913334	IGHV4-j*02	22	5.6	κ	IGKV2-k*03	9	0.30	0.03	3.54	3.29	0.00	0.00	3.38	0.00	0.00
CH505 gp120 wt/d371 diff	913335	IGHV5-a*04	18	4.9	λ	IGLV1-i*02	11	0.04	0.01	3.39	3.21	0.00	0.00	2.65	3.07	2.42
CH505 gp120 wt/d371 diff	913336	IGHV4-g*01	20	6.3	λ	IGLV2-h*01	10	3.85	0.06	3.41	3.35	0.00	0.00	1.04	0.04	0.01
CH505 gp120 wt/d371 diff	913337	IGHV1-a*07	13	2.1	κ	IGKV1-q*02	9	1.09	0.01	3.66	3.01	0.00	0.00	0.02	0.00	0.00
CH505 gp120 wt/d371 diff	913338	IGHV3-ah*02	14	6.6	λ	IGLV3-i*01	11	0.29	0.02	3.63	3.64	3.26	3.34	3.70	3.47	0.00
CH505 gp120 wt/d371 diff	913339	IGHV5-a*04	16	9.4	λ	IGLV1-d*01	11	3.29	0.03	3.54	3.24	0.01	0.02	3.45	3.17	3.20
CH505 gp120 wt/d371 diff	913341	IGHV4-j*03	30	9.3	κ	IGKV1-r*01	9	2.90	0.01	3.18	3.13	0.00	0.00	1.64	0.81	0.00
CH505 gp120 wt/d371 diff	913342	IGHV5-b*03	19	4.9	κ	IGKV1-a*01	9	1.52	0.01	3.48	3.23	0.00	0.00	3.16	0.07	0.00
CH505 gp120 wt/d371 diff	913344	IGHV4-n*03	20	8.2	λ	IGLV2-b*06	10	2.17	0.02	3.53	3.23	0.00	0.00	1.82	0.00	0.00
CH505 gp120 wt/d371 diff	913345	IGHV4-n*03	20	7.9	λ	IGLV2-b*06	10	1.63	0.02	3.47	3.50	0.00	0.01	0.50	0.00	0.00
CH505 gp120 wt/d371 diff	913346	IGHV3-h*02	15	0.3	λ	IGLV1-b*10	11	1.67	0.02	3.39	3.36	0.00	0.00	0.42	0.00	0.00
