# Supplementary material for: IDLV-HIV-1 Env vaccination in non-human primates induces affinity maturation of antigen-specific memory B cells
Source: Commun Biol. 2018 Sep 5;1:134. doi: 10.1038/s42003-018-0131-6 (PMC6125466; doi:10.1038/s42003-018-0131-6)
Supplement: Supplementary file 4 — Supplementary Data 2 [file 42003_2018_131_MOESM4_ESM.docx]

>pGAE-CMV-C.1176gp140Env-Wpre sequence

gatccactagtaacggccgccagtgtgctggaattcggctttgataatcaacctctggattacaaaatttgtgaaagattgactggtattcttaactatgttgctccttttacgctatgtggatacgctgctttaatgcctttgtatcatgctattgcttcccgtatggctttcattttctcctccttgtataaatcctggttgctgtctctttatgaggagttgtggcccgttgtcaggcaacgtggcgtggtgtgcactgtgtttgctgacgcaacccccactggttggggcattgccaccacctgtcagctcctttccgggactttcgctttccccctccctattgccacggcggaactcatcgccgcctgccttgcccgctgctggacaggggctcggctgttgggcactgacaattccgtggtgttgtcggggaagctgacgtcctttccatggctgctcgcctgtgttgccacctggattctgcgcgggacgtccttctgctacgtcccttcggccctcaatccagcggaccttccttcccgcggcctgctgccggctctgcggcctcttccgcgtcttcgccttcgccctcagacgagtcggatctccctttgggccgcctccccgcatcggaagccgaattctgcagatatccatcacactggcggccgctcgagttttataaaagaaaaggggggactggaagggatttattacagtgcaagaagacatagaatcttagacatgtacttagaaaaggaagaaggcatcataccagattggcaggattacacctcaggaccaggaattagatacccaaagacatttggctggctatggaaattagtccctgtaaatgtatcagatgaggcacaggaggatgagaggcattatttaatgcagccagctcaaacttccaagtgggatgacccttggggagaggttctagcgtggaagtttgatccaactctagcctacacttatgaggcatatgctagatacccagaagagttggaagcaagtcaggcctgtcagaactgcatttcgctctgtattcagtcgctctgcggagaggctggcagattgagccctgggaggttctctccagcactagcaggtagagcctgggtgttccctgctagactctcaccagcacttggccagtgctgggcagagtggctccacgcttgcttgcttaaagacctcttcaataaagctgccattttagaagtaagccagtgtgtgttcccatctctcctagtcgccgcctggtcaactcggtactcggtaataagaagaccctggtctgttaggaccctttctgctttgagaaaccgaagcaggaaaatccctagcatggtacccagcttttgttccctttagtgagggttaattccgagcttggcgtaatcatggtcatagctgtttcctgtgtgaaattgttatccgctcacaattccacacaacatacgagccggaagcataaagtgtaaagcctggggtgcctaatgagtgagctaactcacattaattgcgttgcgctcactgcccgctttccagtcgggaaacctgtcgtgccagctgcattaatgaatcggccaacgcgcggggagaggcggtttgcgtattgggcgctcttccgcttcctcgctcactgactcgctgcgctcggtcgttcggctgcggcgagcggtatcagctcactcaaaggcggtaatacggttatccacagaatcaggggataacgcaggaaagaacatgtgagcaaaaggccagcaaaaggccaggaaccgtaaaaaggccgcgttgctggcgtttttccataggctccgcccccctgacgagcatcacaaaaatcgacgctcaagtcagaggtggcgaaacccgacaggactataaagataccaggcgtttccccctggaagctccctcgtgcgctctcctgttccgaccctgccgcttaccggatacctgtccgcctttctcccttcgggaagcgtggcgctttctcatagctcacgctgtaggtatctcagttcggtgtaggtcgttcgctccaagctgggctgtgtgcacgaaccccccgttcagcccgaccgctgcgccttatccggtaactatcgtcttgagtccaacccggtaagacacgacttatcgccactggcagcagccactggtaacaggattagcagagcgaggtatgtaggcggtgctacagagttcttgaagtggtggcctaactacggctacactagaaggacagtatttggtatctgcgctctgctgaagccagttaccttcggaaaaagagttggtagctcttgatccggcaaacaaaccaccgctggtagcggtggtttttttgtttgcaagcagcagattacgcgcagaaaaaaaggatctcaagaagatcctttgatcttttctacggggtctgacgctcagtggaacgaaaactcacgttaagggattttggtcatgagattatcaaaaaggatcttcacctagatccttttaaattaaaaatgaagttttaaatcaatctaaagtatatatgagtaaacttggtctgacagttaccaatgcttaatcagtgaggcacctatctcagcgatctgtctatttcgttcatccatagttgcctgactccccgtcgtgtagataactacgatacgggagggcttaccatctggccccagtgctgcaatgataccgcgagacccacgctcaccggctccagatttatcagcaataaaccagccagccggaagggccgagcgcagaagtggtcctgcaactttatccgcctccatccagtctattaattgttgccgggaagctagagtaagtagttcgccagttaatagtttgcgcaacgttgttgccattgctacaggcatcgtggtgtcacgctcgtcgtttggtatggcttcattcagctccggttcccaacgatcaaggcgagttacatgatcccccatgttgtgcaaaaaagcggttagctccttcggtcctccgatcgttgtcagaagtaagttggccgcagtgttatcactcatggttatggcagcactgcataattctcttactgtcatgccatccgtaagatgcttttctgtgactggtgagtactcaaccaagtcattctgagaatagtgtatgcggcgaccgagttgctcttgcccggcgtcaatacgggataataccgcgccacatagcagaactttaaaagtgctcatcattggaaaacgttcttcggggcgaaaactctcaaggatcttaccgctgttgagatccagttcgatgtaacccactcgtgcacccaactgatcttcagcatcttttactttcaccagcgtttctgggtgagcaaaaacaggaaggcaaaatgccgcaaaaaagggaataagggcgacacggaaatgttgaatactcatactcttcctttttcaatattattgaagcatttatcagggttattgtctcatgagcggatacatatttgaatgtatttagaaaaataaacaaataggggttccgcgcacatttccccgaaaagtgccacctgggaaattgtaaacgttaatattttgttaaaattcgcgttaaatttttgttaaatcagctcattttttaaccaataggccgaaatcggcaaaatcccttataaatcaaaagaatagaccgagatagggttgagtgttgttccagtttggaacaagagtccactattaaagaacgtggactccaacgtcaaagggcgaaaaaccgtctatcagggcgatggcccactacgtgaaccatcaccctaatcaagttttttggggtcgaggtgccgtaaagcactaaatcggaaccctaaagggagcccccgatttagagcttgacggggaaagccggcgaacgtggcgagaaaggaagggaagaaagcgaaaggagcgggcgctagggcgctggcaagtgtagcggtcacgctgcgcgtaaccaccacacccgccgcgcttaatgcgccgctacagggcgcgtcgcgccattcgccattcaggctgcgcaactgttgggaagggcgatcggtgcgggcctcttcgctattacgccagctggcgaaagggggatgtgctgcaaggcgattaagttgggtaacgccagggttttcccagtcacgacgttgtaaaacgacggccagtgaattgtaatacgactcactatagggcgaattggagctccaccgcggtggcggccgctctagaattcccattgcatacgttgtatccatatcataatatgtacatttatattggctcatgtccaacattaccgccatgttgacattgattattgactagttattaatagtaatcaattacggggtcattagttcatagcccatatatggagttccgcgttacataacttacggtaaatggcccgcctggctgaccgcccaacgacccccgcccattgacgtcaataatgacgtatgttcccatagtaacgccaatagggactttccattgacgtcaatgggtggagtatttacggtaaactgcccacttggcagtacatcaagtgtatcatatgccaagtacgccccctattgacgtcaatgacggtaaatggcccgcctggcattatgcccagtacatgaccttatgggactttcctacttggcagtacatctacgtattagtcatcgctattaccatggtgatgcggttttggcagtacatcaatgggcgtggatagcggtttgactcacggggatttccaagtctccaccccattgacgtcaatgggagtttgttttggcaccaaaatcaacgggactttccaaaatgtcgtaacaactccgccccattgacgcaaatgggcggtaggcgtgtacggtgggaggtctatataagcagagctcgtttagtgaaccgcagtcgctctgcggagaggctggcagattgagccctgggaggttctctccagcactagcaggtagagcctgggtgttccctgctagactctcaccagcacttggccagtgctgggcagagtggctccacgcttgcttgcttaaagacctcttcaataaagctgccattttagaagtaagccagtgtgtgttcccatctctcctagtcgccgcctggtcaactcggtactcggtaataagaagaccctggtctgttaggaccctttctgctttgagaaaccgaagcaggaaaatccctagcagattggcgcccgaacaggacttgaaggagagtgagagactcctgagtacggctgagtgaaggcagtaagggcggcaggaaccaaccacgacggagtgctcctataaaggcgcgggtcggtaccagacggcgtgaggagcgggagaggaggaggcctccggttgcaggtaagtgcaacacaaaaaagaaatagctgtcttgttatccaggaagggataataagatagagtgggagatgggcgcgagaaactccgtcttgtcagggaagaaagcagatgaattgaaaaaattattaatcgcatgaattttaaaagaaggggaggaataggggatatgactccagcagaaagattaattaacatgatcactacagaacaagaaatacaatttcaacaatcaaaaaactcaaaatttaaaaattttcgggtctattacagagctcacgcgtgattggagttgggagattataaattagtagagatcactccgattggcttggcccccacagatgtgaagaggtacactactggtggcacctcaagaaataaaagaggggtctttgtgctagggttcttgggttttctcgcaacggcaggttctgcaatgggcgcggcgtcgttgacgctgaccgctcagtcccggactttattggctgggatagtgcagcaacagcaacagctgttggacgtggtcaagagacaacaagaattgttgcgactgaccgtctggggaacaaagaacctccagactagggtcactgccatcgagaagtacttaaaggaccaggcgcagctaaatgcttggggatgtgcgtttagacaagtctgccacactactgtaccatggccaaatgcaagtctaacaccagactggaacaatgatacttggcaagagtgggagcgaaaggttgacttcttggaggaaaatataacagccctcctagaagaggcacaaattcaacaagagaagaacatgtatgaattacaaaagttgaatagctgggatgtgtttggcaattggtttgaccttgcttcttggataaagtatatacaatatggaatttatgtagttgtaggagtaatactgttaagaatagtgatctatatagtacaaatgctagctaagttaaggcaggggtataggccagtgttctcttccccaccctcttatttccagtagactcatacccaacaggacccggcactgccaaccagagaaggcaaagaaggagacggtggagaaggcggtggatcctattatcgatgaattcgagctcggtacccggggatcctctagttattaatagtaatcaattacggggtcattagttcatagcccatatatggagttccgcgttacataacttacggtaaatggcccgcctggctgaccgcccaacgacccccgcccattgacgtcaataatgacgtatgttcccatagtaacgccaatagggactttccattgacgtcaatgggtggagtatttacggtaaactgcccacttggcagtacatcaagtgtatcatatgccaagtacgccccctattgacgtcaatgacggtaaatggcccgcctggcattatgcccagtacatgaccttatgggactttcctacttggcagtacatctacgtattagtcatcgctattaccatggtgatgcggttttggcagtacatcaatgggcgtggatagcggtttgactcacggggatttccaagtctccaccccattgacgtcaatgggagtttgttttggcaccaaaatcaacgggactttccaaaatgtcgtaacaactccgccccattgacgcaaatgggcggtaggcgtgtacggtgggaggtctatataagcagagctctctggctaactagagaacccactgcttactggcttatcgaaattaatacgactcactatagggagacccaagctggctagcgtttaaacgggccctctagcgtcgacaccatgcgcgccaagggcatcctgcgcaactaccagcagtggtggatctggggcatcctgggcttctggatgctgatggtgtgcaacgtggtgggcaacctgtgggtgaccgtgtactacggcgtgcccgtgtggaaggaggccaagaccaccctgttctgcgcctccgacgccaaggcctacgagaaggaggtgcacaacgtgtgggccacccacgcctgcgtgcccaccgaccccaacccccaggagatggtgctggagaacgtgaccgagaacttcaacatgtggaagaacgacatggtggaccagatgcacgaggacgtgatctccctgtgggaccagtccctgaagccctgcgtgaagctgacccccctgtgcgtgaccctgacctgcaccaacaccaccgtgtccaacggctcctccaactccaacgccaacttcgaggagatgaagaactgctccttcaacgccaccaccgagatcaaggacaagaagaagaacgagtacgccctgttctacaagctggacatcgtgcccctgaacaactcctccggcaagtaccgcctgatcaactgcaacacctccgccatcgcccaggcctgccccaaggtgaccttcgagcccatccccatccactactgcgcccccgccggctacgccatcctgaagtgcaacaacaagaccttcaacggcaccggcccctgcaacaacgtgtccaccgtgcagtgcacccacggcatcaagcccgtggtgtccacccagctgctgctgaacggctccctggccgagaaggagatcatcatccgctccgagaacctgaccaacaacgccaagaccatcatcatccacttcaacgagtccgtgggcatcgtgtgcacccgcccctccaacaacacccgcaagtccatccgcatcggccccggccagaccttctacgccaccggcgacatcatcggcgacatccgccaggcccactgcaacgtgtccaagcagaactggaaccgcaccctgcagcaggtgggccgcaagctggccgagcacttccccaaccgcaacatcaccttcaaccactcctccggcggcgacctggagatcaccacccactccttcaactgccgcggcgagttcttctactgcaacacctccggcctgttcaacggcacctaccaccccaacggcacctacaacgagaccgccgtgaactcctccgacaccatcaccctgcagtgccgcatcaagcagatcatcaacatgtggcaggaggtgggccgcgccatgtacgccccccccatcgccggcaacatcacctgcaactccaccatcaccggcctgctgctgacccgcgacggcggcatcaaccagaccggcgaggagatcttccgccccggcggcggcgacatgcgcgacaactggcgcaacgagctgtacaagtacaaggtggtggagatcaagcccctgggcatcgcccccaccaaggccaaggagcgcgtggtggagcgcgagaaggaggccgtgggcatcggcgccgtgttcctgggcttcctgggcgccgccggctccaccatgggcgccgcctccatcaccctgaccgtgcaggcccgccagctgctgtccggcatcgtgcagcagcagtccaacctgctgcgcgccatcgaggcccagcagcacatgctgcagctgaccgtgtggggcatcaagcagctgcaggcccgcgtgctggccatcgagcgctacctgaaggaccagcagctgctgggcatctggggctgctccggcaagctgatctgcaccaccaacgtgccctggaactcctcctggtccaaccgctcccaggaggacatctggaacaacatgacctggatggagtgggagcgcgagatcgacaactacacccacaccatctactccctgctggaggagtcccagatccagcaggagaagaacgagaaggacctgctggccctggactcctggcagaacctgtggtcctggttctccatcaccaagtggctgtggtacatcaagggcctgaacgacatcttcgaggcccagaagatcgagtggcacgagtagg

>pCAGG-NJ-G sequence

gtcgacattgattattgactagttattaatagtaatcaattacggggtcattagttcatagcccatatatggagttccgcgttacataacttacggtaaatggcccgcctggctgaccgcccaacgacccccgcccattgacgtcaataatgacgtatgttcccatagtaacgccaatagggactttccattgacgtcaatgggtggactatttacggtaaactgcccacttggcagtacatcaagtgtatcatatgccaagtacgccccctattgacgtcaatgacggtaaatggcccgcctggcattatgcccagtacatgaccttatgggactttcctacttggcagtacatctacgtattagtcatcgctattaccatgggtcgaggtgagccccacgttctgcttcactctccccatctcccccccctccccacccccaattttgtatttatttattttttaattattttgtgcagcgatgggggcggggggggggggggcgcgcgccaggcggggcggggcggggcgaggggcggggcggggcgaggcggagaggtgcggcggcagccaatcagagcggcgcgctccgaaagtttccttttatggcgaggcggcggcggcggcggccctataaaaagcgaagcgcgcggcgggcgggagtcgctgcgttgccttcgccccgtgccccgctccgcgccgcctcgcgccgcccgccccggctctgactgaccgcgttactcccacaggtgagcgggcgggacggcccttctcctccgggctgtaattagcgcttggtttaatgacggctcgtttcttttctgtggctgcgtgaaagccttaaagggctccgggagggccctttgtgcgggggggagcggctcggggggtgcgtgcgtgtgtgtgtgcgtggggagcgccgcgtgcggcccgcgctgcccggcggctgtgagcgctgcgggcgcggcgcggggctttgtgcgctccgcgtgtgcgcgaggggagcgcggccgggggcggtgccccgcggtgcgggggggctgcgaggggaacaaaggctgcgtgcggggtgtgtgcgtgggggggtgagcagggggtgtgggcgcggcggtcgggctgtaacccccccctgcacccccctccccgagttgctgagcacggcccggcttcgggtgcggggctccgtgcggggcgtggcgcggggctcgccgtgccgggcggggggtggcggcaggtgggggtgccgggcggggcggggccgcctcgggccggggagggctcgggggaggggcgcggcggccccggagcgccggcggctgtcgaggcgcggcgagccgcagccattgccttttatggtaatcgtgcgagagggcgcagggacttcctttgtcccaaatctggcggagccgaaatctgggaggcgccgccgcaccccctctagcgggcgcgggcgaagcggtgcggcgccggcaggaaggaaatgggcggggagggccttcgtgcgtcgccgcgccgccgtccccttctccatctccagcctcggggctgccgcagggggacggctgccttcgggggggacggggcagggcggggttcggcttctggcgtgtgaccggcggctctagagcctctgctaaccatgttcatgccttcttctttttcctacagctccgggcaacgtgctggttgttgtgctgtctcatcattttggcaaagaattcgagctcatcgatgcatggtacccgggcatgctcgaggtcgacacgcgtcaatatgttgtcttatctaatctttgcacttgccgtttcgcccattttgggcaaaattgaaattgtgtttcctcaacataccactggggattggaagagagttccccatgaatataattattgccctaccagcgcagacaagaactcacatgggactcaaacaggaatccctgttgagttaacaatgccaaaaggactaacaacccatcaagttgaaggatttatgtgtcactcagccttgtggatgaccacttgtgacttcagatggtatgggccaaaatacataacccattccatacataatgaagagcctacagattatcaatgtttggaggccattaagtcatacaaagatggagtcagtttcaatccagggtttcctcctcagagctgcgggtatggcacagttaccgatgccgaagcccatattgtgacagttactccccactctgtcaaagtggacgagtacacgggggaatggatcgatccacatttcatcggaggaaggtgcaaaggacaaatttgtgaaacagtccataattccacaaaatggtttacgtcctctgatggagaaagtgtctgcagtcaattgtttactttggttggaggaatttttttctctgattcagaagagattacctccatggggttaccagaaacaggaatcagaagtaattacttcccctacatatctacagagggaatttgcaaaatgccgttttgcagaaaacaggggtacaagcttaaaaatgacctctggttccagatcatggacccagacctggataaaacggttagagatctccctcatattaaggactgtgacctctcctcgtccataatcacaccaggagaacatgctacagacatctcactgatatcagatgttgaaaggatcctggactatgctctttgtcagaatacatggagtaaaattgaatcgggagaaccaattactccggtagatctcagctatcttgggccaaaaaacccaggggttgggccggtcttcaccatcattaacggttccctgcattattttacatcgaagtatctgcgagtcgaattagaaagtcctgtcatacccagaatggaaggaaaagttgcaggaactaggattgtacggcaattgtgggatcagtggtttcctttcggagaagttgagattggacccaatggtgtgttgaaaacgaagcaagggtataaattcccactacacatcattggaactggagaagtagacagtgacatcaaaatggaaagggttgtcaagcactgggaacacccccatattgaggccgctcagacatttttaaaaaaagatgacacaggagaagtcctttattatggcgacaccggagtgtcgaaaaatccagttgaattagtcgagggatggtttagtggatggaggagctccctcatgggagtgctggctgtgattataggatttgtgattttaatgtttttaattaaattgattggagtcttatctagccttttcagacctaaacgcaggccaatctacaaatcagacgtggaaatggctcatttccgttaagctagcagatctttttccctctgccaaaaattatggggacatcatgaagccccttgagcatctgacttctggctaataaaggaaatttattttcattgcaatagtgtgttggaattttttgtgtctctcactcggaaggacatatggagggcaaatcatttaaaacatcagaatgagtatttggtttagagtttggcaacatatgccatatgctggctgccatgaacaaaggtggctataaagaggtcatcagtatatgaaacagccccctgctgtccattccttattccatagaaaagccttgacttgaggttagattttttttatattttgttttgtgttatttttttctttaacatccctaaaattttccttacatgttttactagccagatttttcctcctctcctgactactcccagtcatagctgtccctcttctcttatgaagatccctcgacctgcagcccaagcttggcgtaatcatggtcatagctgtttcctgtgtgaaattgttatccgctcacaattccacacaacatacgagccggaagcataaagtgtaaagcctggggtgcctaatgagtgagctaactcacattaattgcgttgcgctcactgcccgctttccagtcgggaaacctgtcgtgccagcggatccgcatctcaattagtcagcaaccatagtcccgcccctaactccgcccatcccgcccctaactccgcccagttccgcccattctccgccccatggctgactaattttttttatttatgcagaggcgaggccgcctcggcctctgagctattccagaagtagtgaggaggcttttttggaggctaggcttttgcaaaaagctaacttgtttattgcagcttataatggttacaaataaagcaatagcatcacaaatttcacaaataaagcatttttttcactgcattctagttgtggtttgtccaaactcatcaatgtatcttatcatgtctggatccgctgcattaatgaatcgccaacgcgcggggagaggcggtttgcgtattgggcgctcttccgcttcctcgctcactgactcgctgcgctcggtcgttcggctgcggcgagcggtatcagctcactcaaaggcggtaatacggttatccacagaatcaggggataacgcaggaaagaacatgtgagcaaaaggccagcaaaaggccaggaaccgtaaaaaggccgcgttgctggcgtttttccataggctccgcccccctgacgagcatcacaaaaatcgacgctcaagtcagaggtggcgaaacccgacaggactataaagataccaggcgtttccccctggaagctccctcgtgcgctctcctgttccgaccctgccgcttaccggatacctgtccgcctttctcccttcgggaagcgtggcgctttctcaatgctcacgctgtaggtatctcagttcggtgtaggtcgttcgctccaagctgggctgtgtgcacgaaccccccgttcagcccgaccgctgcgccttatccggtaactatcgtcttgagtccaacccggtaagacacgacttatcgccactggcagcagccactggtaacaggattagcagagcgaggtatgtaggcggtgctacagagttcttgaagtggtggcctaactacggctacactagaaggacagtatttggtatctgcgctctgctgaagccagttaccttcggaaaaagagttggtagctcttgatccggcaaacaaaccaccgctggtagcggtggtttttttgtttgcaagcagcagattacgcgcagaaaaaaaggatctcaagaagatcctttgatcttttctacggggtctgacgctcagtggaacgaaaactcacgttaagggattttggtcatgagattatcaaaaaggatcttcacctagatccttttaaattaaaaatgaagttttaaatcaatctaaagtatatatgagtaaacttggtctgacagttaccaatgcttaatcagtgaggcacctatctcagcgatctgtctatttcgttcatccatagttgcctgactccccgtcgtgtagataactacgatacgggagggcttaccatctggccccagtgctgcaatgataccgcgagacccacgctcaccggctccagatttatcagcaataaaccagccagccggaagggccgagcgcagaagtggtcctgcaactttatccgcctccatccagtctattaattgttgccgggaagctagagtaagtagttcgccagttaatagtttgcgcaacgttgttgccattgctacaggcatcgtggtgtcacgctcgtcgtttggtatggcttcattcagctccggttcccaacgatcaaggcgagttacatgatcccccatgttgtgcaaaaaagcggttagctccttcggtcctccgatcgttgtcagaagtaagttggccgcagtgttatcactcatggttatggcagcactgcataattctcttactgtcatgccatccgtaagatgcttttctgtgactggtgagtactcaaccaagtcattctgagaatagtgtatgcggcgaccgagttgctcttgcccggcgtcaatacgggataataccgcgccacatagcagaactttaaaagtgctcatcattggaaaacgttcttcggggcgaaaactctcaaggatcttaccgctgttgagatccagttcgatgtaacccactcgtgcacccaactgatcttcagcatcttttactttcaccagcgtttctgggtgagcaaaaacaggaaggcaaaatgccgcaaaaaagggaataagggcgacacggaaatgttgaatactcatactcttcctttttcaatattattgaagcatttatcagggttattgtctcatgagcggatacatatttgaatgtatttagaaaaataaacaaataggggttccgcgcacatttccccgaaaagtgccacctg

>pAd-SIV-D64V sequence

gaattccatttaaatggaattcgctagcatcatcaataatataccttattttggattgaagccaatatgataatgagggggtggagtttgtgacgtggcgcggggcgtgggaacggggcgggtgacgtagtagtgtggcggaagtgtgatgttgcaagtgtggcggaacacatgtaagcgacggatgtggcaaaagtgacgtttttggtgtgcgccggtgtacacaggaagtgacaattttcgcgcggttttaggcggatgttgtagtaaatttgggcgtaaccgagtaagatttggccattttcgcgggaaaactgaataagaggaagtgaaatctgaataattttgtgttactcatagcgcgtaatatttgtctagggagatctatgtatcttatcatgtctggatccccgcggccgctctagaactagtggatccgggccgccaccgcggtggagctccaaatatcgcagtttcgatataggtgacagacgatatgaggctatatcgccgatagaggcgacatcaagctggcacatggccaatgcatatcgatctatacattgaatcaatattggccattagccatattattcattggttatatagcataaatcaatattggctattggccattgcatacgttgtatccatatcataatatgtacatttatattggctcatgtccaacattaccgccatgttgacattgattattgactagttattaatagtaatcaattacggggtcattagttcatagcccatatatggagttccgcgttacataacttacggtaaatggcccgcctggctgaccgcccaacgacccccgcccattgacgtcaataatgacgtatgttcccatagtaacgccaatagggactttccattgacgtcaatgggtggagtatttacggtaaactgcccacttggcagtacatcaagtgtatcatatgccaagtacgccccctattgacgtcaatgacggtaaatggcccgcctggcattatgcccagtacatgaccttatgggactttcctacttggcagtacatctacgtattagtcatcgctattaccatggtgatgcggttttggcagtacatcaatgggcgtggatagcggtttgactcacggggatttccaagtctccaccccattgacgtcaatgggagtttgttttggcaccaaaatcaacgggactttccaaaatgtcgtaacaactccgccccattgacgcaaatgggcggtaggcgtgtacggtgggaggtctatataagcagagctcgtcgaggccgcctggaagcttgccctccggttgcaggtaagtgcaacacaaaaaagaaatagctgtcttgttatccaggaagggataataagatagagtgggagatgggcgcgagaaactccgtcttgtcagggaagaaagcagatgaattagaaaaaattaggctacgacccggcggaaagaaaaagtacatgttgaagcatgtagtatgggcagcaaatgaattagatagatttggattagcagaaagcctgttggagaacaaagaaggatgtcaaaaaatactttcggtcttagctccattagtgccaacaggctcagaaaatttaaaaagcctttataatactgtctgcgtcatctggtgcattcacgcagaagagaaagtgaaacacactgaggaagcaaaacagatagtgcagagacacctagtggtggaaacaggaacagcagaaactatgccaaaaacaagtagaccaacagcaccatctagcggcagaggaggaaattacccagtacaacaaataggtggtaactatgtccacctgccattaagcccgagaacattaaatgcctgggtaaaattgatagaggaaaagaaatttggagcagaagtagtgccaggatttcaggcactgtcagaaggctgcaccccctatgacattaatcagatgttaaattgtgtgggagaccatcaagcggctatgcagattatcagagatattataaatgaggaggctgcagattgggacttgcagcacccacaaccagctccacaacaaggacagcttagggagccgtcaggatcagatattgcaggaacaactagttcagtagatgaacaaatccagtggatgtacagacaacagaaccccataccagtaggcaacatttacaggagatggatccaactggggttgcaaaaatgtgtcagaatgtataacccaacaaacattctagatgtaaaacaagggccaaaagagccatttcagagctatgtagacaggttctacaaaagcttaagagcagaacaaacagatgcagcagtaaagaattggatgactcaaacactgctgattcaaaatgctaacccagattgcaagctagtgctgaaggggctgggtgtgaatcccaccctagaagaaatgctgacggcttgtcaaggagtagggggaccaggacagaaggctagattaatggcagaagccctgaaagaggccctcgcaccagtgccaatcccttttgcagcagcccagaagaggggaccaagaaagccaattaagtgttggaattgtgggaaggagggacactctgcaaggcaatgcagagccccaagaagacagggatgctggaaatgtggaaaaatggaccatgttatggccaaatgcccagacagacaggcgggttttttaggccttggtccatggggaaagaagccccgcaatttccccatggctcaagtgcatcaggggctgacgccaactgctcccccagaggacccagctgtggatctgctaaagaactacatgcagttgggcaagcagcagagagaaagcagagagaagccttacaaggaggtgacagaggatttgctgcacctcaattctctctttggaggagaccagtagtcactgctcatattgaaggacagcctgtagaagtattattggatacaggggctgatgattctattgtaacaggaatagagttaggtccacattataccccaaaaatagtaggaggaataggaggttttattaatactaaagaatacaaaaatgtaaaaatagaagttttaggcaaaaggattaaagggacaatcatgacaggggacactccgattaacatttttggtaggaatttgctaacagctctggggatgtctctaaatcttcccatagctaaggtagagcctgtaaaagtcaccttaaagccaggaaaggttggaccaaaattgaagcagtggccattatcaaaagaaaagatagttgcattaagagaaatctgtgaaaagatggaaaaggatggtcagttggaggaagctcccccgaccaatccatacaacacccccacatttgccataaagaaaaaagataagaacaaatggagaatgctgatagattttagggaactaaatagggtcactcaggactttacagaagtccaattaggaataccacaccctgcaggactagcaaaaaggaaaaggattacagtactggatataggtgatgcatatttctccatacctctagatgaagaatttaggcagtacactgcctttactttaccatcagtaaataatgcagagccaggaaaacgatacatttataaggttctgcctcagggatggaaggggtcaccagccatcttccaatacactatgagacatgtgctagaacccttcaggaaggcaaatccagatgtgaccttagtccagtatatggatgacatcttaatagctagtgacaggacagacctggaacatgacagggtagttttacagctaaaggaactcttaaatagcatagggttctctaccccagaagagaaattccaaaaagatcccccatttcaatggatggggtacgaattgtggccgacaaaatggaagttgcaaaagatagagttgccacaaagagagacctggacagtgaatgatatacagaagttagtaggagtattaaattgggcagctcaaatttatccaggtataaaaaccaaacatctctgtaggttaattagaggaaaaatgactctaacagaggaagttcagtggactgagatggcagaagcagaatatgaggaaaataagataattctcagtcaggaacaagaaggatgttattaccaagaaggcaagccattagaagccacggtaataaagagtcaggacaatcagtggtcttataaaattcaccaagaagacaaaatactgaaagtaggaaaatttgcaaagataaagaatacacataccaatggagttagactattagcacatgtaatacagaaaataggaaaggaagcaatagtgatctggggacaggtcccaaaattccacttaccagttgagagggatgtatgggaacagtggtggacagactattggcaggtaacctggataccggagtgggattttatctcaacgccaccactagtaagattagtcttcaatctagtgaaggaccctatagagggagaagaaacctattatacagatggatcatgtaataaacagtcaaaagaagggaaagcaggatatatcacagataggggcaaagacaaagtaaaagtgttagaacagactactaatcaacaagcagaattagaagcatttctcatggcattgacagactcagggccaaagacaaatattatagtagattcacaatatgttatgggaataataacaggatgccctacagaatcagagagcaggctagttaaccaaataatagaagaaatgattaaaaagtcagaaatttatgtagcatgggtaccagcacacaaaggtataggaggaaaccaagaaatagaccacctagttagtcaggggattagacaagttctcttcttggaaaagatagagccagcacaagaagaacatgataaataccatagtaatgtaaaagaattggtattcaaatttggattacccagaatagtggccagacagatagtagacacctgtgataaatgtcatcagaaaggagaagctatacatgggcaggtaaattcagatctagggacttggcaaatggtatgtacccatctagaaggaaaaatagtcatagttgcagtacatgtagctagtggattcatagaagcagaagtaattccacaagagacaggaagacagacagcactatttctgttaaaattggcaggcagatggcctattacacatctacacacagataatggtgctaactttgcctcgcaagaagtaaagatggttgcatggtgggcagggatagagcacacctttggggtaccatacaatccacagagtcagggagtagtggaagcaatgaatcaccacctgaaaaatcaaatagatagaatcagggaacaagcaaattcagtagaaaccatagtattaatggcagttcattgcatgaattttaaaagaaggggaggaataggggatatgactccagcagaaagattaattaacatgatcactacagaacaagaaatacaatttcaacaatcaaaaaactcaaaatttaaaaattttcgggtctattacagagaaggcagagatcaactgtggaagggacccggtgagctattgtggaaaggggaaggagcagtcatcttaaaggtagggacagacattaaggtagtacccagaagaaaggctaaaattatcaaagattatggaggaggaaaagaggtggatagcagttcccacatggaggataccggagaggctagagaggtggcatagcctcataaaatatctgaaatataaaactaaagatctacaaaaggtttgctatgtgccccattttaaggtcggatgggcatggtggacctgcagcagagtaatcttccccctacaggaaggaagccatttagaagtacaagggtattggcatttgacaccagaaagagggtggctcagtacttatgcagtgaggataacctggtactcaaggaacttttggacagatgtaacaccagactatgcagacattttactgcatagcacttatttcccttgctttacagcgggagaagtgagaagggccatcaggggagaacaactgctgtcttgctgcaagttcccgagagctcataggtaccaggtaccaagcctacagtacttagcactaaaagtagtaagcgatgtcagatcccagggagagaatcccacctggaaacagtggagaagagacaataggagaggccttcgaatggctaaacagaacagtagaggagataaacagagaggcagtaaaccacctaccaagggagctgattttccaggtttggcaaaggtcttgggaatactggcatgatgaacaagggatgtcacaaagctatgtaaaatacagatacttgtgtttaatgcaaaaggctttatttatgcattgcaagaaaggctgtagatgtctaggggaaggacacggggcaggaggatggagaccaggacctcctcctcctccccctccaggactagcataaatggaagaaagacctccagaaaatgaaggcccacaaagggaaccatgggatgaatgggtagtggaggttctggaagaattgaaagaagaagctttaaaacattttgatcctcgcttgctaactgcacttggtaatcatatctataatagacatggagacacccttgagggagcaggagaactcattagaatcctccaacgagcgctcttcatgcattttagaggcggatgcaaccactccagaatcggccaacctgggggaggaaatcctctctcaactataccgccctcttgaggcgtgctataacacatgctattgtaaaaagtgttgctaccattgccagttttgttttcttaaaaagggattggggatatgttatgagcagtcacgaaagagaagaagaactccgaaaaaggctaaggctaatacatcttctgcatcaaacaagtaagtatgccattgcatacgttgtatccatatcataatatgtacatttatattggctcatgtccaacattaccgccatgttgacattgattattgactagtatcaccatgagtgcagaggtggcagaactgtatcgattggagttgggagattataaattagtagagatcactccgattggcttggcccccacagatgtgaagaggtacactactggtggcacctcaagaaataaaagaggggtctttgtgctagggttcttgggttttctcgcaacggcaggttctgcaatgggcgcggcgtcgttgacgctgaccgctcagtcccggactttattggctgggatagtgcagcaacagcaacagctgttggacgtggtcaagagacaacaagaattgttgcgactgaccgtctggggaacaaagaacctccagactagggtcactgccatcgagaagtacttaaaggaccaggcgcagctaaatgcttggggatgtgcgtttagacaagtctgccacactactgtaccatggccaaatgcaagtctaacaccagactggaacaatgatacttggcaagagtgggagcgaaaggttgacttcttggaggaaaatataacagccctcctagaagaggcacaaattcaacaagagaagaacatgtatgaattacaaaagttgaatagctgggatgtgtttggcaattggtttgaccttgcttcttggataaagtatatacaatatggaatttatgtagttgtaggagtaatactgttaagaatagtgatctatatagtacaaatgctagctaagttaaggcaggggtataggccagtgttctcttccccaccctcttatttccagtagactcatacccaacaggacccggcactgccaaccagagaaggcaaagaaggagacggtggagaaggcggtggcaacagctcctggccttggcagatagaatatattcatttcctgatccgccaactgatacgcctcttgacttggctattcagcaactgcagaaccttgctatcgagagcataccagatcctccaaccaatactccagaggctctctgcgaccctacgaagggttcgagaagtcctcaggactgaactgacctacctacaatatgggtggagctatttccatgaggcggtccaagccggctggagatctgcgacagaaactcttgcgggcgcgtggagagacttatgggagactcttaggagaggtggaagatggatcctcgcaatccctagagatcataatcagccataccacatttgtagaggttttacttgctttaaaaaacctcccacacctccccctgaacctgaaacataaaatgaatgcaattgttgttgttaacttgtttattgcagcttataatggttacaaataaagcaatagcatcacaaatttcacaaataaagcatttttttcactgcattctagttgtggtttgtccaaactcatcaatgtatcttatcatgtctggatcgaattcgatatcaagcttatcgatgatatcagatctggaaggtgctgaggtacgatgagacccgcaccaggtgcagaccctgcgagtgtggcggtaaacatattaggaaccagcctgtgatgctggatgtgaccgaggagctgaggcccgatcacttggtgctggcctgcacccgcgctgagtttggctctagcgatgaagatacagattgaggtactgaaatgtgtgggcgtggcttaagggtgggaaagaatatataaggtgggggtcttatgtagttttgtatctgttttgcagcagccgccgccgccatgagcaccaactcgtttgatggaagcattgtgagctcatatttgacaacgcgcatgcccccatgggccggggtgcgtcagaatgtgatgggctccagcattgatggtcgccccgtcctgcccgcaaactctactaccttgacctacgagaccgtgtctggaacgccgttggagactgcagcctccgccgccgcttcagccgctgcagccaccgcccgcgggattgtgactgactttgctttcctgagcccgcttgcaagcagtgcagcttcccgttcatccgcccgcgatgacaagttgacggctcttttggcacaattggattctttgacccgggaacttaatgtcgtttctcagcagctgttggatctgcgccagcaggtttctgccctgaaggcttcctcccctcccaatgcggtttaaaacataaataaaaaaccagactctgtttggatttggatcaagcaagtgtcttgctgtctttatttaggggttttgcgcgcgcggtaggcccgggaccagcggtctcggtcgttgagggtcctgtgtattttttccaggacgtggtaaaggtgactctggatgttcagatacatgggcataagcccgtctctggggtggaggtagcaccactgcagagcttcatgctgcggggtggtgttgtagatgatccagtcgtagcaggagcgctgggcgtggtgcctaaaaatgtctttcagtagcaagctgattgccaggggcaggcccttggtgtaagtgtttacaaagcggttaagctgggatgggtgcatacgtggggatatgagatgcatcttggactgtatttttaggttggctatgttcccagccatatccctccggggattcatgttgtgcagaaccaccagcacagtgtatccggtgcacttgggaaatttgtcatgtagcttagaaggaaatgcgtggaagaacttggagacgcccttgtgacctccaagattttccatgcattcgtccataatgatggcaatgggcccacgggcggcggcctgggcgaagatatttctgggatcactaacgtcatagttgtgttccaggatgagatcgtcataggccatttttacaaagcgcgggcggagggtgccagactgcggtataatggttccatccggcccaggggcgtagttaccctcacagatttgcatttcccacgctttgagttcagatggggggatcatgtctacctgcggggcgatgaagaaaacggtttccggggtaggggagatcagctgggaagaaagcaggttcctgagcagctgcgacttaccgcagccggtgggcccgtaaatcacacctattaccgggtgcaactggtagttaagagagctgcagctgccgtcatccctgagcaggggggccacttcgttaagcatgtccctgactcgcatgttttccctgaccaaatccgccagaaggcgctcgccgcccagcgatagcagttcttgcaaggaagcaaagtttttcaacggtttgagaccgtccgccgtaggcatgcttttgagcgtttgaccaagcagttccaggcggtcccacagctcggtcacctgctctacggcatctcgatccagcatatctcctcgtttcgcgggttggggcggctttcgctgtacggcagtagtcggtgctcgtccagacgggccagggtcatgtctttccacgggcgcagggtcctcgtcagcgtagtctgggtcacggtgaaggggtgcgctccgggctgcgcgctggccagggtgcgcttgaggctggtcctgctggtgctgaagcgctgccggtcttcgccctgcgcgtcggccaggtagcatttgaccatggtgtcatagtccagcccctccgcggcgtggcccttggcgcgcagcttgcccttggaggaggcgccgcacgaggggcagtgcagacttttgagggcgtagagcttgggcgcgagaaataccgattccggggagtaggcatccgcgccgcaggccccgcagacggtctcgcattccacgagccaggtgagctctggccgttcggggtcaaaaaccaggtttcccccatgctttttgatgcgtttcttacctctggtttccatgagccggtgtccacgctcggtgacgaaaaggctgtccgtgtccccgtatacagacttgagaggcctgtcctcgaccgatgcccttgagagccttcaacccagtcagctccttccggtgggcgcggggcatgactatcgtcgccgcacttatgactgtcttctttatcatgcaactcgtaggacaggtgccggcagcgctctgggtcattttcggcgaggaccgctttcgctggagcgcgacgatgatcggcctgtcgcttgcggtattcggaatcttgcacgccctcgctcaagccttcgtcactggtcccgccaccaaacgtttcggcgagaagcaggccattatcgccggcatggcggccgacgcgctgggctacgtcttgctggcgttcgcgacgcgaggctggatggccttccccattatgattcttctcgcttccggcggcatcgggatgcccgcgttgcaggccatgctgtccaggcaggtagatgacgaccatcagggacagcttcaaggatcgctcgcggctcttaccagcctaacttcgatcactggaccgctgatcgtcacggcgatttatgccgcctcggcgagcacatggaacgggttggcatggattgtaggcgccgccctataccttgtctgcctccccgcgttgcgtcgcggtgcatggagccgggccacctcgacctgaatggaagccggcggcacctcgctaacggattcaccactccaagaattggagccaatcaattcttgcggagaactgtgaatgcgcaaaccaacccttggcagaacatatccatcgcgtccgccatctccagcagccgcacgcggcgcatctcgggcagcgttgggtcctggccacgggtgcgcatgatcgtgctcctgtcgttgaggacccggctaggctggcggggttgccttactggttagcagaatgaatcaccgatacgcgagcgaacgtgaagcgactgctgctgcaaaacgtctgcgacctgagcaacaacatgaatggtcttcggtttccgtgtttcgtaaagtctggaaacgcggaagtcagcgccctgcaccattatgttccggatctgcatcgcaggatgctgctggctaccctgtggaacacctacatctgtattaacgaagcctttctcaatgctcacgctgtaggtatctcagttcggtgtaggtcgttcgctccaagctgggctgtgtgcacgaaccccccgttcagcccgaccgctgcgccttatccggtaactatcgtcttgagtccaacccggtaagacacgacttatcgccactggcagcagccactggtaacaggattagcagagcgaggtatgtaggcggtgctacagagttcttgaagtggtggcctaactacggctacactagaaggacagtatttggtatctgcgctctgctgaagccagttaccttcggaaaaagagttggtagctcttgatccggcaaacaaaccaccgctggtagcggtggtttttttgtttgcaagcagcagattacgcgcagaaaaaaaggatctcaagaagatcctttgatcttttctacggggtctgacgctcagtggaacgaaaactcacgttaagggattttggtcatgagattatcaaaaaggatcttcacctagatccttttaaattaaaaatgaagttttaaatcaatctaaagtatatatgagtaaacttggtctgacagttaccaatgcttaatcagtgaggcacctatctcagcgatctgtctatttcgttcatccatagttgcctgactccccgtcgtgtagataactacgatacgggagggcttaccatctggccccagtgctgcaatgataccgcgagacccacgctcaccggctccagatttatcagcaataaaccagccagccggaagggccgagcgcagaagtggtcctgcaactttatccgcctccatccagtctattaattgttgccgggaagctagagtaagtagttcgccagttaatagtttgcgcaacgttgttgccattgctgcaggcatcgtggtgtcacgctcgtcgtttggtatggcttcattcagctccggttcccaacgatcaaggcgagttacatgatcccccatgttgtgcaaaaaagcggttagctccttcggtcctccgatcgttgtcagaagtaagttggccgcagtgttatcactcatggttatggcagcactgcataattctcttactgtcatgccatccgtaagatgcttttctgtgactggtgagtactcaaccaagtcattctgagaatagtgtatgcggcgaccgagttgctcttgcccggcgtcaacacgggataataccgcgccacatagcagaactttaaaagtgctcatcattggaaaacgttcttcggggcgaaaactctcaaggatcttaccgctgttgagatccagttcgatgtaacccactcgtgcacccaactgatcttcagcatcttttactttcaccagcgtttctgggtgagcaaaaacaggaaggcaaaatgccgcaaaaaagggaataagggcgacacggaaatgttgaatactcatactcttcctttttcaatattattgaagcatttatcagggttattgtctcatgagcggatacatatttgaatgtatttagaaaaataaacaaataggggttccgcgcacatttccccgaaaagtgccacctgacgtctaagaaaccattattatcatgacattaacctataaaaataggcgtatcacgaggccctttcgtcttcaa
